# Drivers of polar bear behavior and the possible effects of prey availability on foraging strategy

**DOI:** 10.1186/s40462-022-00351-4

**Published:** 2022-11-16

**Authors:** Ron R. Togunov, Andrew E. Derocher, Nicholas J. Lunn, Marie Auger-Méthé

**Affiliations:** 1 Institute for the Oceans and Fisheries, The University of British Columbia, V6T 1Z4 Vancouver, Canada; 2 Department of Zoology, The University of British Columbia, Vancouver, V6T 1Z4 Canada; 3 Department of Biological Sciences, University of Alberta, Edmonton, T6G 2E9 Canada; 4 Wildlife Research Division, Science and Technology Branch, Environment and Climate Change Canada, Edmonton, T6G 2E9 Canada; 5 Department of Statistics, The University of British Columbia, Vancouver, V6T 1Z4 Canada

**Keywords:** Polar bear, Behavior, Foraging strategy, Optimal foraging, Telemetry, Hidden Markov Model

## Abstract

**Background:**

Change in behavior is one of the earliest responses to variation in habitat suitability. It is therefore important to understand the conditions that promote different behaviors, particularly in areas undergoing environmental change. Animal movement is tightly linked to behavior and remote tracking can be used to study ethology when direct observation is not possible.

**Methods:**

We used movement data from 14 polar bears (*Ursus maritimus*) in Hudson Bay, Canada, during the foraging season (January–June), when bears inhabit the sea ice. We developed an error-tolerant method to correct for sea ice drift in tracking data. Next, we used hidden Markov models with movement and orientation relative to wind to study three behaviors (stationary, area-restricted search, and olfactory search) and examine effects of 11 covariates on behavior.

**Results:**

Polar bears spent approximately 47% of their time in the stationary drift state, 29% in olfactory search, and 24% in area-restricted search. High energy behaviors occurred later in the day (around 20:00) compared to other populations. Second, olfactory search increased as the season progressed, which may reflect a shift in foraging strategy from still-hunting to active search linked to a shift in seal availability (i.e., increase in haul-outs from winter to the spring pupping and molting seasons). Last, we found spatial patterns of distribution linked to season, ice concentration, and bear age that may be tied to habitat quality and competitive exclusion.

**Conclusions:**

Our observations were generally consistent with predictions of the marginal value theorem, and differences between our findings and other populations could be explained by regional or temporal variation in resource availability. Our novel movement analyses and finding can help identify periods, regions, and conditions of critical habitat.

**Supplementary Information:**

The online version contains supplementary material available at 10.1186/s40462-022-00351-4.

## Background

Animals exhibit a broad diversity of behaviors to meet their needs for survival, growth, and reproduction. Each behavior has consequences to the individual and has distinct relationships with the external environment [112, 163]. Changes in behavior and their associated movement patterns may represent the earliest measurable response to variation in habitat suitability and potential effects of environmental change [16, 164]. For example, optimal foraging theory and the marginal value theorem predict that time spent in a resident state increases with patch quality [21, 100, 119]. Understanding the spatial and environmental determinants of animal behavior is central to ecology and conservation. Identifying the factors that promote different behaviors may be particularly important in areas experiencing rapid environmental change or where animals occupy the limits of their realized niche. In these areas, shifts in occupied states or time budgets may precede other indicators of habitat quality (e.g., body condition, reproductive success, or survival; [16, 28, 164]). For example, Alaskan moose (*Alces alces*) exhibit strongest selection for denser canopy cover at the northern and southern limits of their range [67], and tropical songbirds exhibit strongest intraspecific territoriality closer to the limit of their altitudinal range [66].

The Arctic is warming at several times the global average, resulting in reduced sea-ice extent and a prolonged ice-free period [76, 80, 103, 130, 150]. The reductions in sea ice have caused a shift toward smaller primary and secondary producers [29, 167], and negatively affected Arctic fish [24, 98] and pinnipeds [56, 62, 136]. Polar bears (*Ursus maritimus*) rely on sea ice as a platform to access their primary prey, ringed seals (*Pusa hispida*) and bearded seals (*Erignathus barbatus*), as well as for reproduction and travel [45, 48, 140, 148]. Reduction in sea ice has led to an increase in the energetic cost of travel. Greater habitat fragmentation has increased polar bear path tortuosity [9], more open water has increased the frequency of long-distance swimming events [105, 115], and increased ice drift speed has increased the cost of station-keeping [5, 34, 90]. Further, polar bears have exhibited shifts in distribution [85], reduced access to prey [45, 147, 160], a longer fasting period [127], increased exposure to zoonotic pathogens [116], higher levels of cortisol [13], reduced body condition [127, 144], reduced access to denning habitat [96, 127], reduced reproduction [146], and consequently reduced abundance in several populations [14, 87, 101, 123, 124]. The ecological effects of climate change on polar bears are population specific. For example, a group of polar bears near the southern limit of the East Greenland subpopulation offset reduced sea ice by using a glacial mélange for year-round access to prey [82]. In addition, in parts of the high Arctic, increasing temperatures may have led to a regime shift from historically unproductive multi-year ice to more productive seasonal pack ice and consequently stable or increasing polar bear populations (e.g., M’Clintock Channel and Gulf of Boothia; [36, 37]). Many of the effects of climate change on polar bears are associated with behavioral shifts including changes in foraging [47], migration [107, 115], and denning strategies [38, 102].

**Fig. 1:**
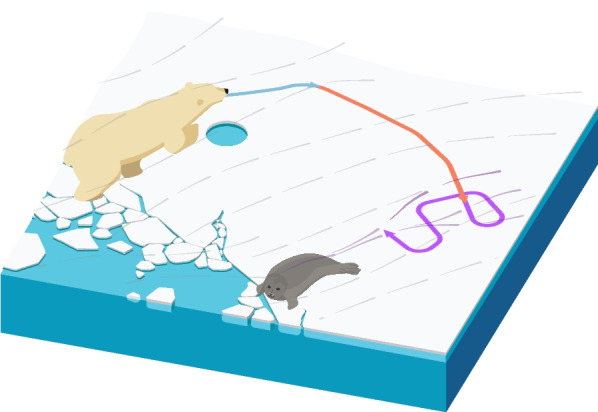
Illustration of polar bear behavior and movement. Polar bear track drifting $\approx 15^{\circ }$ relative to wind (gray) when stationary on sea ice (e.g., when still-hunting by breathing hole; blue), moving $\approx 90^{\circ }$ relative to wind during olfactory search (red) to maximize probability of encountering odor plumes (purple), and random movement relative to wind during area-restricted search (purple track). Reproduced with permission from [155]

Polar bears exhibit a diversity of behaviors to successfully exploit the spatiotemporally dynamic sea-ice habitat (Fig. 1; [104, 138, 161]). In areas of seasonal sea ice, polar bears migrate between the terrestrial refugia and on-ice foraging grounds [11, 22]. They exhibit philopatry to their summering grounds and compensate for sea ice motion in their navigation and for station keeping [5, 34, 74, 90]. Polar bears rely both on visual and olfactory search to hunt sparsely distributed prey [133, 138], which may be influenced by presence of daylight [152, 153]. However, the expansive and remote nature of their habitat impedes behavioral research. Direct observational research (e.g., [65, 138]) is limited in spatial or temporal extent. Insight into polar bear ecology across larger spatiotemporal scales often relies on remote tracking data, however these studies are typically not behaviorally-explicit (e.g., [35, 75, 81, 92]). Although there have been a few large-scale multi-behavioral studies using polar bear telemetry (e.g., [4, 107, 161]), these were either limited to two simple behavioral states (e.g., active or inactive) or did not investigate associations between behaviors and the environment.

Recent advances in data acquisition [99] and analytical methods (e.g., [94, 155]) have enabled the identification of more intricate behaviors and research at an increasingly large scale and resolution. Pagano et al. [104] described the use of accelerometers to identify up to ten fine-scale behaviors, and Pagano et al. [107] used a combination of accelerometer data and conductivity sensors to identify resting, walking, and swimming. Unfortunately, most existing telemetry datasets do not lend themselves to many of the newer analytical methods because they lack necessary auxiliary data (i.e., they only estimate tag location). However, Togunov et al. [155] described the use of location data in combination with wind to identify up to three behavioral states, a method readily applicable to most existing polar bear movement data.

Using remote tracking data for behavioral research is further complicated by sea ice motion. The motion of sea ice is imparted on the polar bear track, changes the apparent speed and orientation, and complicates behavior characterization and classification [5, 50, 155]. The conventional method used to remove sea ice motion is to subtract satellite-based estimates of ice velocity from the movement track (e.g., [5, 10, 74]), however, this approach assumes there is negligible bias in the sea ice motion data, which is typically not the case [31, 154, 165]. Rather than correcting for drift, it is often more accurate to integrate the motion of the environment into the movement model (e.g., [69, 94, 155]). McClintock and Michelot [94] and Johnson et al. [69] described observed movement as a trade-off between directional persistence and bias toward wind or ocean currents. However, because environmental flow affects the animal’s movement, the observed track does not reflect the voluntary movement/orientation of the animal. Similarly, Togunov et al. [155] identified periods where the bear was stationary and passively drifting on the sea ice by describing the movement as a random walk with bias toward a constant angle relative to wind (Fig. 1). However, ice motion affects the movement of non-stationary behaviors, which was not accounted for in Togunov et al. [155] and may lead to misclassification. One of the fundamental challenges of identifying voluntary movement is that both observed bearing and movement speed depend on animal direction and speed, such that a change in either animal speed or direction influences both the observed step length and turning angle (see Appendix D). To our understanding, there is no statistical movement model that can simultaneously estimate parameters of behavior-specific movement and environment-induced motion.

Given climate change induced alterations of sea ice, we were interested in understanding how polar bears change their behavior in space and time and how these behaviors were affected by environmental variability. Our objectives were to: (1) develop a sea ice motion correction model that accounts for error in satellite-based estimates, (2) examine behavior time budgets during the winter foraging period, (3) identify factors associated with different behaviors, and (4) examine broad-scale and behavior-specific habitat use.

## Methods

### Study area and telemetry data

**Fig. 2:**
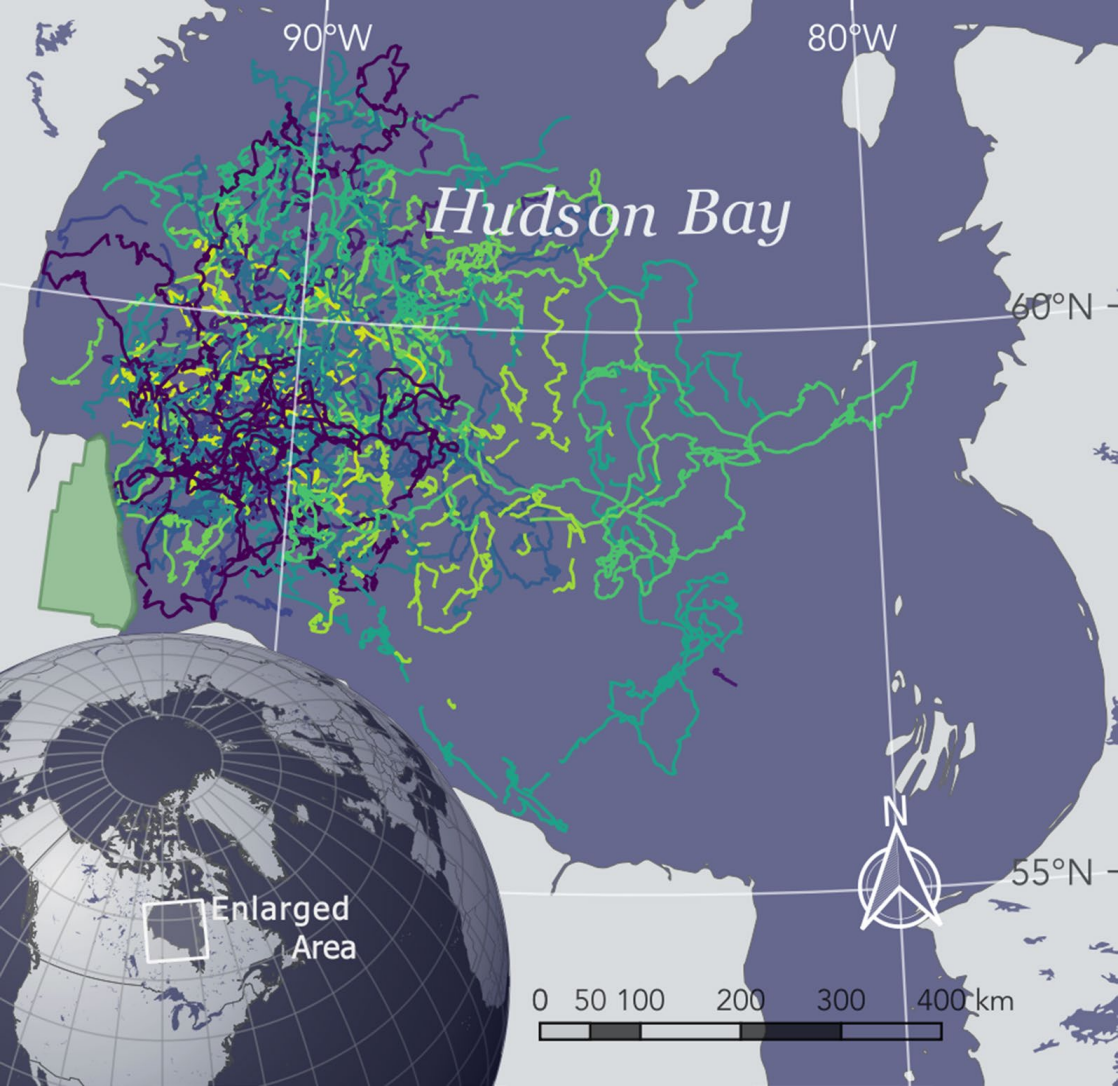
Hudson Bay study area (enlarged) and polar bear tracks (colored lines). Gaps in telemetry data $>6$ h and bouts $<24$ h were excluded from analysis and are not shown. The green region represents Wapusk National Park

Hudson Bay, Canada is a large inland sea with an area of 830,000 km$^2$ (Fig. 2; [118]). The Bay is characterized by seasonally-present sea ice, which begins to form during freeze-up in late November. The ice covers the majority of the Bay from January to May and is comprised primarily of drifting pack ice and a 10–15 km wide fringe of land-fast ice [42, 83]. The sea ice concentration begins to decline in May, typically reaching 50% cover in early July, and melting completely by early August [30, 129]. The focal period of this study was January to July, representing the primary feeding and mating period and the subsequent decline in sea ice (i.e., break-up).

As part of a long-term study of the population ecology of polar bears in Western Hudson Bay [87, 122, 123, 146], 107 adult females with cubs were tranquilized from helicopters [145] during the summers of 2010–2019 and equipped with Argos^®^ or Iridium^®^ satellite-linked global positioning system (GPS) collars (Telonics, Mesa, AZ). Lone females were not collared as they may have been pregnant and would remain in maternity dens up to seven months after collaring. Males were not collared because their neck circumference is greater than that of their head and would not retain collars. Tagging was performed primarily in the Western Hudson Bay polar bears’ summering and denning grounds in Wapusk National Park, Manitoba (Fig. 2; [43, 126, 139]). To determine age, a vestigial premolar was extracted from each bear whose age was unknown (i.e., bears $>1.5$ years that were not previously captured), and age was determined by counting annuli in the cementum [19]. The animal handling protocols performed were approved by the University of Alberta Animal Care and Use Committee for Biosciences (permit numbers: AUP00000033 and AUP00003667) and by the Environment Canada Prairie and Northern Region Animal Care Committee.

The collars were programmed to last two years after which they would release on a predefined date. Of the 107 collars deployed, 79 obtained locations every 4 h, 25 collars obtained locations every 2 h, and three collars obtained locations every 30 min. A preliminary analysis indicated that the 4-h collars could reliably only identify two behaviors (data not shown). To identify at least three behaviors, we used only the collars at 2-h or 30-min frequency, and we sampled the 30-min data to a 2-h frequency. The bear locations were projected into a Universal Transverse Mercator coordinate reference system (NAD83 Teranet Ontario Lambert, EPSG: 5321).

We used hidden Markov models (HMM) to investigate the relationship of different behavioral states in relation to environmental covariates. The classic HMM assumes the observation data is in discrete time and that there is no missing data in the predictor variables [94, 168]. To avoid interpolating large gaps, we segmented the location data into separate bouts whenever missing locations spanned more than 6 h (i.e., we interpolated a maximum of three missing locations). To remove data-sparse bouts, we removed segments spanning < 24 h or those with fewer than eight locations (as in [155]). Any missing locations in the remaining bouts were interpolated using the R Package crawl [70, 71]. The remaining telemetry data used in the subsequent analysis are presented in Fig. 5.

Next, we annotated this telemetry data with the following covariates: bear age and cub status from when the bears were tagged; ordinal date and hour of the GPS fix; sun altitude calculated using the R package oce [72]; wind velocity, snow depth, total precipitation, surface solar radiation from the ERA5 meteorological reanalysis project [59]; sea ice concentration [27]; bathymetry [63]; tidal currents [135]; and sea ice motion vectors by the National Snow and Ice Data Center (NSIDC; [156, 157]). Spatial covariates provided as a gridded raster were interpolated in space and time as in Togunov et al. [152, 153]. Hypotheses being tested and their associated predictions as well as detailed description of each covariate (i.e., source, spatial and temporal resolution, and method of interpolation) are presented in Appendix A.

### Sea ice motion correction

Between 2005 and 2015, 20 GPS collars originally deployed on polar bears had been dropped by the bears (or were on bears that died) on the sea ice, collectively yielding 10,409 locations during the months of December–July [154]. To correct for sea ice motion in the telemetry data, without assuming the environmental data is free of bias, we first fitted a biased correlated random walk (BCRW; all notation used in this paper is described in Table 1) model to tracks of these passively drifting collars [154]. This BCRW described the motion of the dropped collars as a function of either ERA5 wind velocities [59] or NSIDC sea ice motion vectors [156, 157]. The data source (i.e., ERA5 wind or NSIDC sea ice motion) that predicted sea ice motion was used for the correction. In this instance, the hourly wind data outperformed the daily NSIDC data (details in Additional file 1: Appendix C). Next, we used the BCRW fitted to the dropped collars to predict and subtract the estimated component of sea ice motion driven by wind from the bear telemetry data (details in Additional file 1: Appendix B). After correcting for wind-advection, the 2-h bear collars still retained motion from tidal currents, which complete $360^{\circ }$ counter-clockwise rotations approximately every 12 h [135]. This tidal motion could not be corrected using the dropped collars due to their lower 4-h resolution [154]. Therefore, the residual tidal current was integrated into the motion of the drift state in the behavioral model (details in Sect. 2.3). Although it is challenging to remove sea ice motion from the movement track, we expected reducing its effect would increase the accuracy of the HMM—particularly for characterizing states with movement speed similar to sea-ice speed. Appendix D compares the results of the ice motion-correction method implemented in this paper to no ice motion-correction, a correction using satellite-derived sea ice motion vectors, and a method that integrates (rather than subtracting) sea ice motion into all behaviors.

### Behavior analysis

Some behaviors exhibit orientation bias relative to the external stimuli, which is defined as taxis [26]. The degree of bias can occur along a spectrum from being primarily governed by bias relative to external stimuli (e.g., biased random walk, BRW, advective correlated velocity model, CVM), to a trade-off between directional persistence and orientation bias (e.g., BCRW), to being primarily governed by directional persistence (e.g., CRW, unbiased CVM; [8, 25, 26, 55]). Bias toward an angle relative to a stimulus is defined as menotaxis. The ability to detect and quantify movement bias depends on the sampling interval (e.g., measurement of location or acceleration) as well as the statistical approach used to model movement [55]. For example, low-frequency data may lack information on movement bias, and conversely, high-frequency data may have a large degree of autocorrelation that may obscure information on bias [55, 155]. In addition to sampling frequency, sampling regularity and measurement error can impact the efficacy of various statistical approaches in describing biological processes [54, 55, 132, 155].

We used an adapted version of the HMM with menotactic behaviors described in Togunov et al. [155] to investigate three movement behaviors: drifting, area-restricted search (*ARS*), and olfactory search. We described these behaviors using a four-state HMM in which the drifting and *ARS* behaviors were represented by their own respective states (*D* and *ARS*) and olfactory search was divided into two discrete states corresponding to bias left and bias right relative to wind ($O^{(L)}$ and $O^{(R)}$, respectively; Fig. 1; [155]). Under this HMM framework, the states were assumed to be a discrete-time latent Markovian process, where the probability of a state $S_t$ at time $t \in 1,\ldots , T$ depends only on the state at the previous time $S_{t-1}$, and the observed data ${\mathbf {X}}_t$ depends only on the hidden state $S_t$ [95, 168]. The state transition probabilities $\gamma _{i,j} = {\mathrm {Pr}}(S_{t+1} = j| S_{t} = i)$ for $i,j \in \{1,\ldots ,N\}$ (where *N* is the number of states) are summarized by the $N \times N$ transition probability matrix, $\mathbf {\Gamma }$. We extracted two variables from the tracking data: step length $l_t \in (0, \infty )$ (the distance between consecutive locations) and turning angle $\phi _t \in (-\pi ,\pi ]$ (change in bearing between consecutive steps; [84, 94, 155]). Following Togunov et al. [155], we assumed step lengths followed a Gamma distribution:

1 $$\begin{aligned} l_{S,t} \sim {{\mathrm {gamma}}}(\mu _{S,t}^{(l)}, \sigma _{S,t}^{(l)}), \end{aligned}$$

where $\mu _{S,t}^{(l)} > 0$ and $\sigma _{S,t}^{(l)} > 0$ are the state-specific mean and standard deviation, respectively, of the step length at time *t* [94, 155]. The motion of the drift state was defined as a function of predicted tidal currents as it could not be corrected for using the 4-h dropped collars. Specifically, we defined the mean step length following:

2 $$\mu _{{S,t}}^{{(l)}} = \left\{ {\begin{array}{*{20}l} {\beta _{{1,S}} + \beta _{2} r_{t}^{{(tide)}} } \hfill & {\quad {\text{if}}\,\,S = D} \hfill \\ {\beta _{{1,S}} } \hfill & {\quad {\text{Otherwise}},} \hfill \\ \end{array} } \right.$$

where $\beta _{1,S} \in (-\infty , \infty )$ is the state-specific intercept coefficient for step length mean and $\beta _{2} \in (-\infty , \infty )$ is a slope coefficient representing how the mean step length of *D* is affected by tidal speed $r^{(tide)}_t$. Further, we used pseudo-design matrices in conjunction with working boundaries to ensure that the step length of olfactory search was faster than ARS and that ARS was faster than drift (i.e., $\mu _{D,t}^{(l)}< \mu _{ARS,t}^{(l)} < \mu _{O^{(L,R)},t}^{(l)}$; details in Additional file 1: Appendix E; [94]).

We assumed the turning angle followed a von Mises distribution:

3 $$\begin{aligned} \phi _{S,t} \sim {\mathrm {vMises}}(\mu _{S,t}^{(\phi )}, \kappa _{S,t}^{(\phi )}), \end{aligned}$$

where $\mu _{S,t}^{(\phi )} \in (-\pi ,\pi ]$ is the state-specific mean turning angle parameter at time *t* and $\kappa _{S,t}^{(\phi )} > 0$ is the state-specific concentration parameter around $\mu _{S,t}^{(\phi )}$ [94, 155]. We assumed the mean turning angle for *D* was a circular-circular regression function of tidal currents and that the degree of bias toward the direction of tides increased with tidal speed (details in Additional file 1: Appendix E). The *ARS* state turning mean angle was fixed at 0 (i.e., $\mu _{ARS}^{(\phi )} = 0$). The olfactory search states were modeled as menotactic BCRWs with biases toward a unknown angles relative to wind. Following Togunov et al. [155], this menotactic BCRWs modeled the mean turning angle as a trade-off between bias parallel to wind and bias perpendicular to wind. The mean turning angle of each behavior was modeled as follows:

4 $$\mu _{{S,t}}^{{(\phi )}} = \left\{ {\begin{array}{*{20}l} {{\text{atan2}}(\alpha _{{1,S}} r_{t}^{{(tide)}} \sin \psi _{t}^{{(tide)}} ,1 + \alpha _{{1,S}} r_{t}^{{(tide)}} \cos \psi _{t}^{{(tide)}} )} \hfill & {\quad {\text{if}}\,\,S = D,} \hfill \\ 0 \hfill & {\quad {\text{if}}\,\,S = ARS,} \hfill \\ {{\text{atan2}}(\alpha _{{1,S}} \sin \psi _{t}^{{(wind)}} - \alpha _{{2,S}} \cos \psi _{t}^{{(wind)}} ,\quad 1 + \alpha _{{1,S}} \cos \psi _{t}^{{(wind)}} + \alpha _{{2,S}} \sin \psi _{t}^{{(wind)}} )} \hfill & {\quad {\text{if}}\,\,S = O^{{(R)}} ,} \hfill \\ {{\text{atan2}}(\alpha _{{1,S}} \sin \psi _{t}^{{(wind)}} + \alpha _{{2,S}} \cos \psi _{t}^{{(wind)}} ,\quad 1 + \alpha _{{1,S}} \cos \psi _{t}^{{(wind)}} - \alpha _{{2,S}} \sin \psi _{t}^{{(wind)}} )} \hfill & {\quad {\text{if}}\,\,S = O^{{(L)}} ,} \hfill \\ \end{array} } \right.$$

where $\psi ^{(wind)}_t \in (-\pi ,\pi ]$ and $\psi ^{(tide)}_t \in (-\pi ,\pi ]$ represent the directions of wind and tides, respectively, at time *t* relative to the track bearing at time $t-1$, $\alpha _{1, S} \in {\mathbb {R}}$ represents the state-specific bias coefficient parallel to $\psi _t$, and $\alpha _{2, S} \in (-\infty , \infty ) \in {\mathbb {R}}$ represents the state-specific bias coefficient toward $90^{\circ }$ anti-clockwise of $\psi _t$ [155]. As $O^{(L)}$ and $O^{(R)}$ represented the same underlying behavior, they shared the parameters for state transition probabilities $\gamma _{i,j}$, step length $\mu _{S,t}^{(l)}$ and $\sigma _{S,t}^{(l)}$, turning angle concentration $\kappa _{S,t}^{(\phi )}$, and bias parallel to wind $\alpha _{1, S}$, and we fixed $\alpha _{2, O^{(R)}} = \alpha _{2, O^{(L)}}$ [155]. Following Togunov et al. [155], the angle of attraction relative to wind was represented by $\vartheta = {\mathrm {atan2}}(\alpha _{2}, \alpha _{1})$. In addition, we could represent where a behavior lies along the spectrum of CRW and BRW using the scaled magnitude of attraction $M^*_S \in [0,1)$:

5 $$\begin{aligned} M^*_S = \frac{\sqrt{\alpha _{1,S}^2 + \alpha _{2,S}^2}}{1 + \sqrt{\alpha _{1,S}^2 + \alpha _{2,S}^2}}. \end{aligned}$$

The CRW and BRW are limiting cases of Eq. 5, where $M^*\rightarrow 0$ and $M^*\rightarrow 1$, respectively, while a BCRW would have an intermediate value of $M^*$. That is, a value of $M^*$ close to 0 represents behavior with high directional persistence, and a value close to 1 represents behavior with orientation highly correlated to an external stimuli.

To investigate how environmental conditions influence the probability of different behaviors, we modeled the state transition probabilities as functions of the annotated covariates [84, 94]. To allow for non-linearity, ordinal date, sun altitude, wind velocity, surface solar radiation, ice concentration were fitted in linear and quadratic form, and hour of the day was fitted as a cosinor model [94]. To determine which form (i.e., linear or quadratic, cosinor) to use, we fitted HMMs with each form of the covariate on their own (i.e., with no other covariates), and selected the form with the lowest Akaike information criterion (AIC; [1]). We assessed the co-linearity among competing covariates (e.g., sun altitude, surface solar radiation, and hour of the day; and ice concentration and ordinal date). For any covariates with a correlation $>0.5$, we selected the covariate with the lowest AIC in the consequent model selection. Each remaining covariate tested unique and non-competing hypotheses regarding polar bear behavior, therefore, any combination of covariates would yield a valid ecological model. Therefore, we used forward and backward AIC model selection to determine which combination of covariates best explained state transitions [17, 111].

Over time, state probabilities of a Markov chain converge to the ‘stationary distribution’, which represent the marginal probability of a state assuming the covariates remain constant [84, 110, 168]. We estimated the 95% confidence interval on the stationary distribution as an indicator of significant within-state change in relation to covariates in state probability or between-state probability following McClintock and Michelot [94]. Because we were not examining variation between bears, we did not include random effect on individual ID, and all estimated model coefficients were shared among individuals [93, 94]. The HMMs were fitted using the R package momentuHMM [94].

To investigate the spatial distribution of states, we first determined the most likely state for each step using the Viterbi algorithm [168]. Second, we rasterized the state-decoded steps by assigning them to cells of a regular 50 km grid. Third, to reduce temporal autocorrelation, we rarefied the steps into unique “bear days”, such that for each cell, we retained only one data point for unique bear ID, date, and state. A simple measure of the most frequent state in a cell would bias states that were more common overall and fail to identify where uncommon states were disproportionately frequent. Therefore, for each cell, we identified which state was most frequent relative to the frequency of each state across all cells. Specifically, for each cell *i*, we defined the state $\varvec{S'}$ as the state with the highest within-cell proportion relative to proportion across all cells following:

6 $$\begin{aligned} {\varvec{S'}}_i = \mathop {\mathrm{argmax}}\limits _{S=1,2,3,4}\left( \frac{N_i^{(S)}/N_i}{N^{(S)}/N}\right) , \end{aligned}$$

where $N_i^{(S)}$ is the number of bear days in cell *i* for state *S*, $N_i$ is the total number of bear days in cell *i* across all states, $N^{(S)}$ is the number of bear days across all cells for state *S*, and *N* is the total number of bear days across all cells and states. $\varvec{S'}$ was calculated for the entire data set, as well as separately for early winter (January–March) and late winter (April–June) to compare seasons, separately for years with low ice concentration and high ice concentration to compare years with different conditions, and separately for younger (6–14 years) to older (15–20 years) adult bears to compare age classes. We obtained weekly sea-ice coverage in Hudson Bay from January to June from Ice Graph version 2.5 [131]. We calculated the mean total ice concentration for each year and classified each into either years with below or above mean total ice concentration. Spatial state segregation was described qualitatively.

In addition to spatial state segregation, we compared the spatial extent of telemetry data by season and between years with high and low concentration. Utilization distribution (UD) was calculated using the 80% autocorrelated kernel density estimator (aKDE) using the R package ctmm [18]; this analysis was only descriptive and did not assess statistical significance. All analyses were conducted in R version 4.1.1 [120].

## Results

We obtained 31550 locations during the focal months of January to June from 14 unique bears. Some collars provided data across multiple years, yielding 24 unique “bear-years”. The movement bouts contained 2484 (7%) missing locations that were interpolated using the R package crawl. After filtering out short and data-sparse bouts from the interpolated segments, we retained 33160 unique steps. Each year had between 2084 (2019) and 10092 (2020) locations, and we obtained between 4199 (February) and 6545 (April) locations across months.

**Fig. 3:**
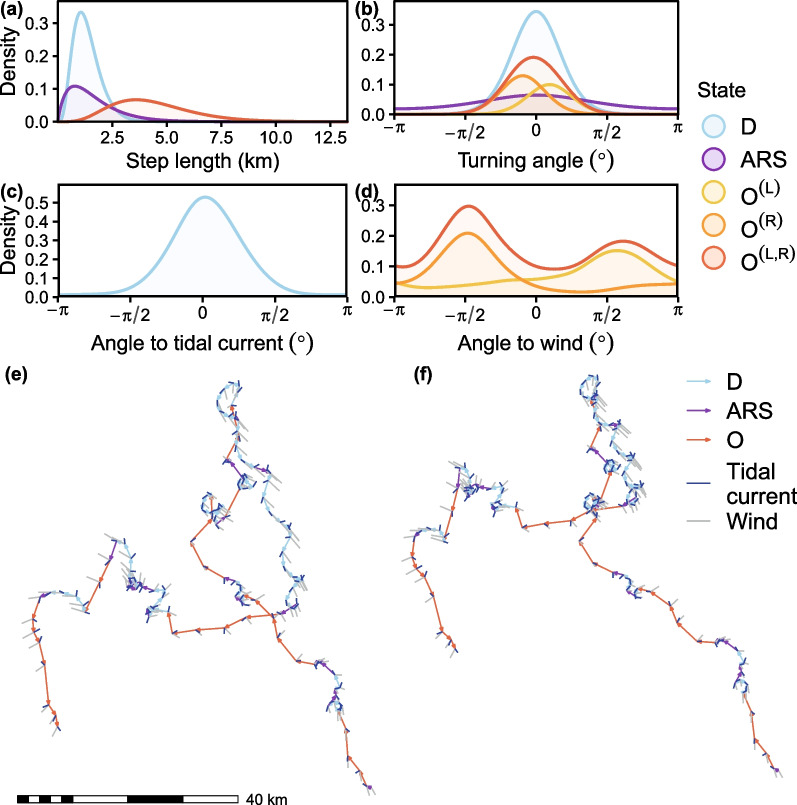
Predicted state characteristics. **a** Step length distribution for each state; **b** turning angle distribution for each state; polar bear orientation relative to **c** tidal currents and **d** wind; and example from one bear bout contrasting the **e** original track and **f** wind-forcing-corrected track. *D* and *ARS* represent drift and area-restricted search, respectively, and *O* represent olfactory search (left, $O^{(L)}$, or right, $O^{(R)}$, relative to wind). Tracks in **e** and **f** are colored by the decoded states and show the estimated wind (gray) and tidal current (blue) velocities. All data (except in panel **e**) were based on the wind-forcing-corrected tracks and the top model fitted to seven years of polar bear telemetry data from Western Hudson Bay, Canada

Both forward and backward model selection converged to the same top model. In this model, drift was characterized as a slow BCRW with a moderate turning bias in the same direction as tidal currents. At the median estimated tidal speed of 0.29 km h$^{-1}$, the mean step length of drift was $0.63 \pm 0.31$ km h$^{-1}$ (${\hat{\mu }}^{(l)}_{D}, {\hat{\sigma }}^{(l)}_{D}$; Table 2, Fig. 3a). The turning angle concentration (${\hat{\kappa }}^{(\phi )}_D = 3.64$; Table 2, Fig. 3b) and scaled magnitude of attraction (${\hat{M}}^*_{O^{(L)},O^{(R)}} = 0.41$; Table 2) were moderate, best characterizing drift as a BCRW. The direction of tidal currents explained much of the variation in direction of drift with the majority of drift direction falling within $5.0^{\circ } \pm 43.8$ SD of the tidal currents (Fig. 3c and f).

ARS was characterized as a slow CRW with no bias relative to wind. The estimated mean step length was $0.76 \pm 0.59$ km h$^{-1}$ (${\hat{\mu }}^{(l)}_{ARS}, {\hat{\sigma }}^{(l)}_{ARS}$; Table 2, Fig. 3a). Mean turning angle was fixed to zero and the turning angle concentration was the lowest among the states (${\hat{\kappa }}^{(\phi )}_{ARS} = 0.62$; Table 2, Fig. 3b), best characterizing ARS as a CRW with low persistence.

Olfactory search was characterized as a fast BCRW with a bias relative to wind. Olfactory search had the highest estimated mean step length (${\hat{\mu }}^{(l)}_{O^{(L)},O^{(R)}} = 2.04$, ${\hat{\sigma }}^{(l)}_{O^{(L)},O^{(R)}} = 0.93$ km h$^{-1}$; Fig. 3a), with an overall bias toward $\pm 92 ^{\circ }$ relative to wind (downwind bias of ${\hat{\alpha }}_{1,O^{(L)},O^{(R)}} -0.011$, and crosswind bias of ${\hat{\alpha }}_{2,O^{(L)},O^{(R)}} \pm 0.303$; Table 2, Fig. 3d). The turning angle concentration was the highest among the states ($\kappa ^{(\phi )}_{O^{(L)},O^{(R)}} = 4.275$; Table 2, Fig. 3b). The scaled magnitude of attraction relative to wind was moderately low (${\hat{M}}^*_{O^{(L)},O^{(R)}} = 0.233$ Table 2), best characterizing olfactory search as a BCRW.

**Fig. 4:**
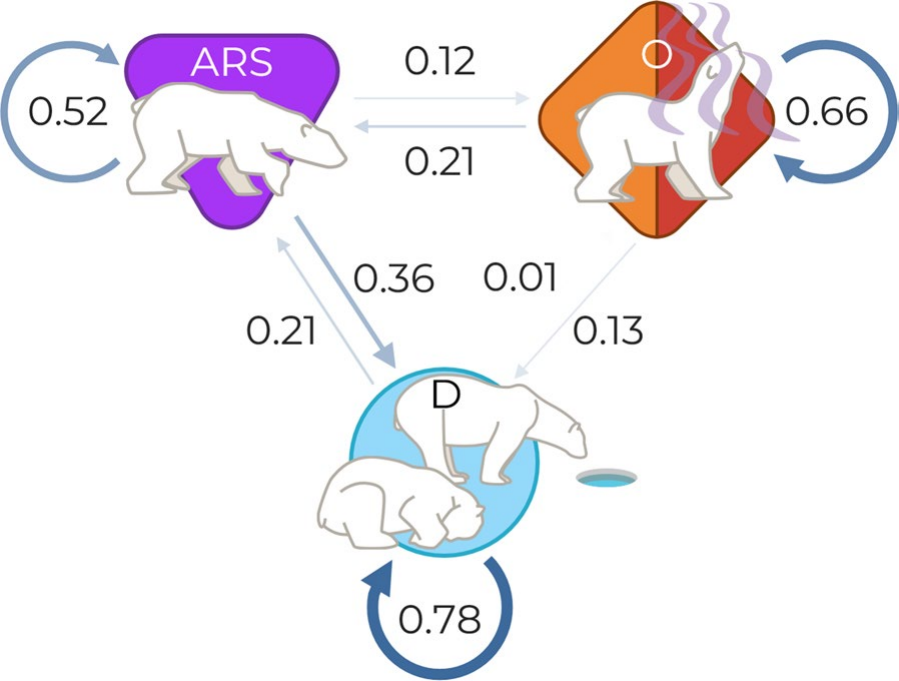
Markov chain depicting state transition probabilities. Arrow thickness and transparency represents weight of transition probability (numeric value) assuming mean values for all covariates. *D* and *ARS* represent drift and area-restricted search, respectively, and *O* represent olfactory search (left, $O^{(L)}$, or right, $O^{(R)}$, relative to wind)

Based on the Viterbi-decoded states, polar bears spent approximately 47% of their time in the drift state, 24% in ARS, and 29% in olfactory search. All behavioral states were most likely to remain within the same state (state transitions between 0.52 in *ARS* to 0.78 in *D*; Fig. 4). $O^{(L,R)}$ was about 1.6 times more likely to transition *ARS* than *D*, *ARS* was about 2.9 times more likely to transition *D* than $O^{(L,R)}$, and *D* was about 19.6 times more likely to transition *ARS* than $O^{(L,R)}$ (Fig. 4).

**Fig. 5:**
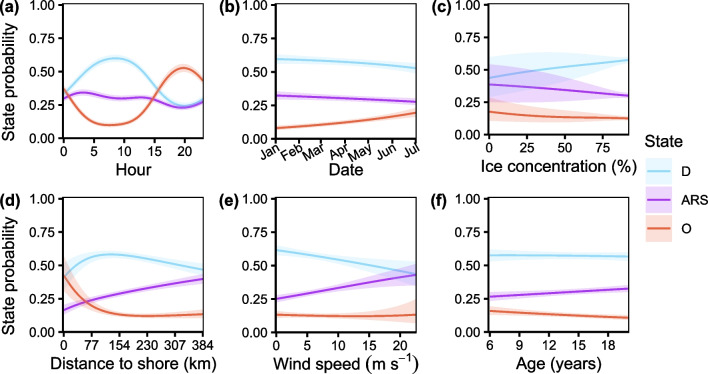
Stationary state probabilities as functions of **a** hour, **b** day, **c** ice concentration, **d** distance to shore, **e** wind speed, and **f** bear age. Shaded areas represent the 95% confidence interval. *D*, *ARS*, and *O* represent drift, area-restricted search, and olfactory search, respectively

Six covariates on the state transition probability were identified in the top model: hour, day, ice concentration, distance to shore, wind speed, and bear age. The highest variability in state probability was with respect to hour of the day. The drift state was most frequent between 00:00 and 15:30 h (local time, UTC -5 H) with a peak around 08:30 h. Olfactory search was most frequent between 15:30 and 00:00 with a peak around 20:00 h. However, this evening peak activity appeared to be primarily driven by movement in January–May, while bears did not appear to exhibit significant diurnal variation in June (Additional file 1: Fig. D1). ARS exhibited little variation compared to drift and olfactory search, though it appeared to decrease from a peak at 03:00 h to a trough at 20:00 h (Fig. 5a). With respect to ordinal date, drift and ARS decreased as the season progressed, while olfactory search increased (Fig. 5b). With respect to ice concentration, the confidence intervals decreased with increasing ice concentration, likely due to the larger amount of data at higher concentrations. ARS and olfactory search marginally decreased with increasing concentration, while drift increased with ice concentration. At ice concentrations $< 50\%$, drift and ARS had similar probabilities (Fig. 5c). The probability of ARS gradually increased as distance to shore increased. The probability of being in a drift state increased up to $\sim 130$ km from shore, then gradually declined. The probability of olfactory search declined rapidly until a distance of $\sim 150$ km from shore, then remained relatively consistent. Near shore, the probabilities of drift and olfactory search were similar, and at a maximum distance of $\sim 390$ km, the probabilities of drift and ARS were similar (Fig. 5d). As wind speed increased, the probability of drift decreased, ARS increased, while olfactory search remained relatively consistent. At the highest observed wind speeds $\sim 20$ m s$^{-1}$, the probabilities of drift and ARS were similar (Fig. 5e). As age increased, the probability of ARS increased, olfactory search decreased, while drift remained relatively consistent (Fig. 5f).

**Fig. 6:**
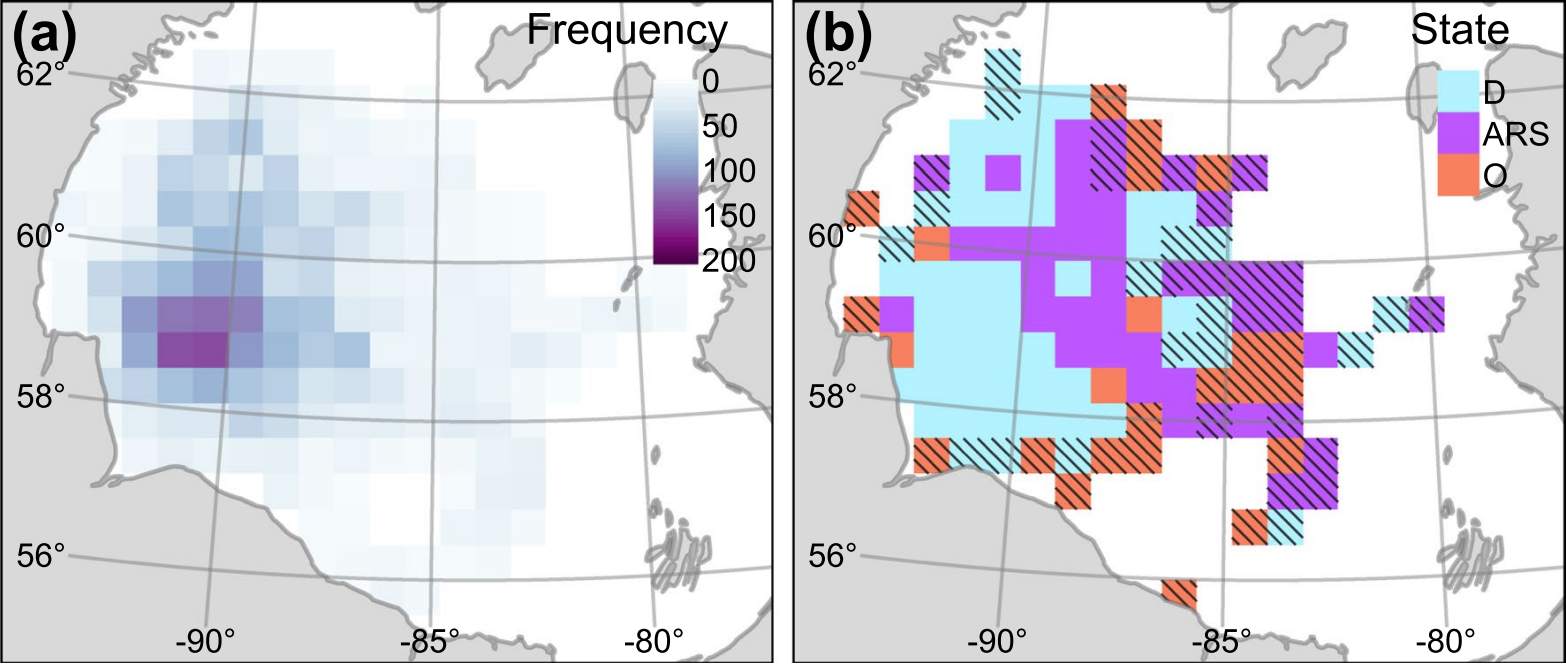
Distribution of predicted states. **a** total number of bear days and **b** state $\varvec{S'}$ with the highest within-cell proportion relative across-cell proportion. Cells with $<7$ bear days were not plotted and cells with $<21$ bear days were hashed

**Fig. 7:**
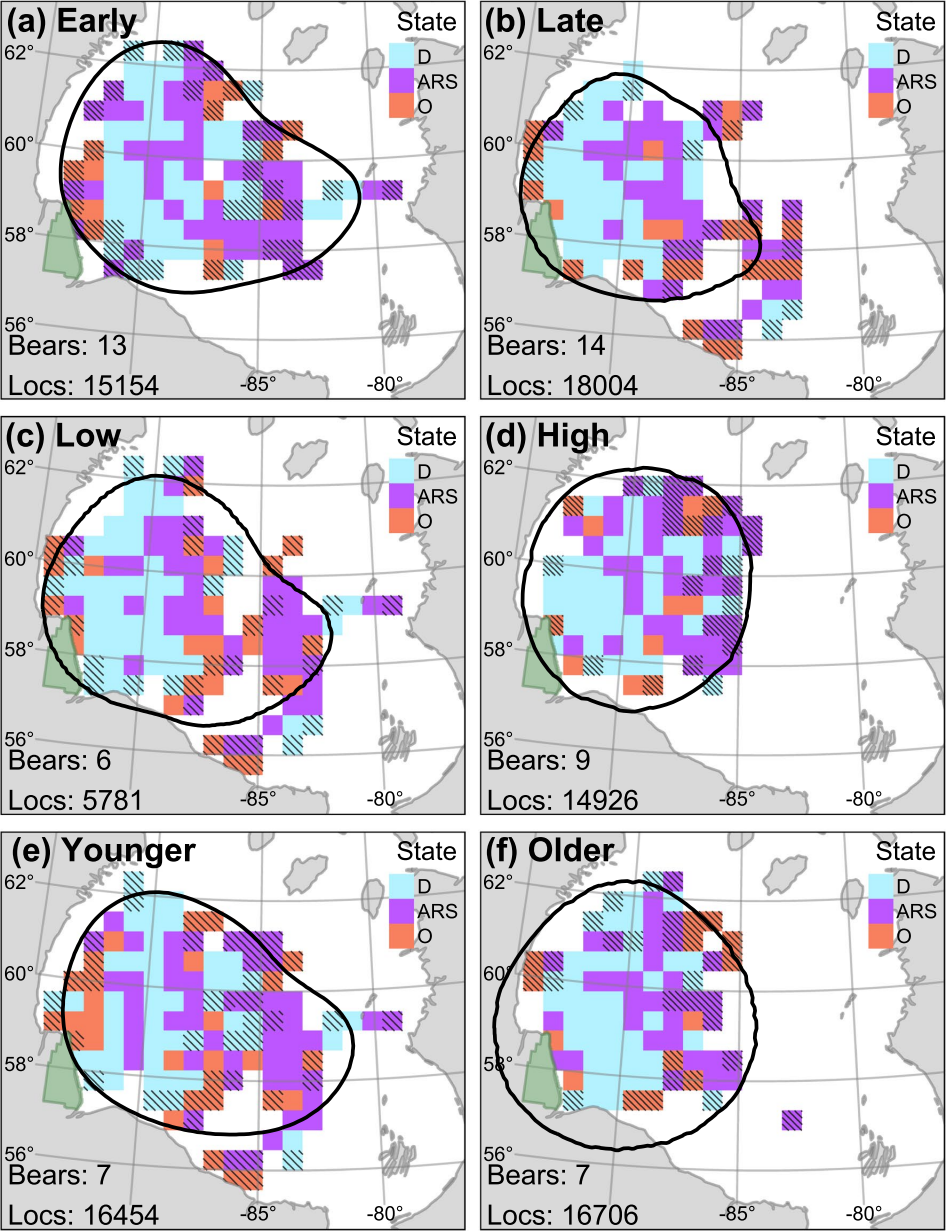
Maps of state $\varvec{S'}$ with the highest within-cell proportion relative to across-cell proportion. Data were subset into **a** early winter (January–March) or **b** late winter (April–June); **c** years with low ice concentration (2011, 2016, 2017, and 2021) or **d** years with high ice concentration (2018, 2019, and 2020); and **e** youngest seven bears (6–14 years) or **f** oldest seven bears (15–20 years). Maps were based on Viterbi-decoded states and rarefied to bear days. Cells with $<7$ bear days were not plotted and cells with $<21$ bear days are hashed. The number of unique bears and number of locations are presented on the bottom left. The green region represents Wapusk National Park. Contour line represents the 80% utilization distribution

There was a non-uniform distribution of location data across the Bay, with the highest concentration of locations occurring around $-90.5^{\circ }$ longitude and $58.5^{\circ }$ latitude (Fig. 6a). There appeared to be marginal segregation of states, with drift appearing to be more common west of $-89^{\circ }$ longitude, ARS was more common east of $-89^{\circ }$ longitude, and olfactory search was more common around the periphery of the overall extent (Fig. 6b). The spatial segregation between drift and ARS was apparent in late season, when mean annual ice concentration was high, and among older bears (Fig. 7). The UD was $\approx 31$% greater during early winter (January–March; 80% UD = 316,550 km$^2$) compared to late winter (April–June; 80% UD = 242,100 km$^2$; Fig. 7a and b). The 80% UD in years with below average ice concentration (2011, 2016, 2017, and 2021; $\mu \pm$SE$=89.03\pm 0.28$) was $\approx 15$% greater (low: 277,753 km$^2$, high: 267,418 km$^2$) compared to years above average ice concentration (2018, 2019, and 2020; $\mu \pm$SE$=91.95\pm 0.31$; Fig. 7c and d). The 80% UD of younger individuals was $\approx 5$% smaller compared to older individuals (younger: 312,822 km$^2$, older: 330,313 km$^2$; Fig. 7e and f).

## Discussion

Polar bears exhibit a high degree of behavioral plasticity and diversity [104, 138, 151, 161], however the remote and dynamic nature of their habitat has made it difficult to study their behavior, particularly during the critical winter foraging period. We used remote tracking data to investigate the spatiotemporal distribution of three movement states representative of three important behaviors (stationary/drifting, area-restricted search, and olfactory search) and to examine what factors may promote them. We identified six factors that appear to affect state probability and the spatial variation in state distribution and segregation. Most notably, we observed diurnal and intra-annual variation that may be indicative of a circadian rhythm and seasonal shifts in foraging strategy corresponding to known changes in prey availability. In addition, we observed variation in the spatial extent of movement that may be related to variation in habitat quality or intraspecific competitive exclusion.

One of the key challenges when applying HMMs to identify behavior from movement data is the biological interpretation of states [95, 117]. We classified movement into three states, each of which may represent more than one behavior. For example, the drift state may represent any stationary behavior, including sheltering during adverse weather, resting, still-hunting, or prey handling. To facilitate the interpretation of the results, we used prior knowledge on the behavior and phenology of polar bears and their prey. In addition, we interpreted states through the lens of classic optimal foraging theory and the marginal value theorem, which provide predictions on relationships between residency times and energy expenditure in relation to resource availability and habitat quality [21, 44, 119].

### State probability

Nearly half of polar bears’ overall time budget was occupied by the sedentary drift behavior, which was also the most frequent state. These results align with previous work revealing that polar bears spend the majority of their time in stationary behaviors [104, 138]. All behaviors were most likely to remain in the same state. After remaining within the same state, olfactory search was most likely to transition to ARS, ARS was most likely to transition to drift, and drift was most likely to transition to ARS. The sequence from olfactory search to ARS, then drift, aligns with the prediction that following successful olfactory search, bears should transition to a scent-localization strategy. If the target is an active breathing hole, bears would transition to still-hunting [106, 138], and if the target is a hauled out seal, bears may attempt to stalk and ambush the seal [133, 138]. If a bear successfully captures a seal, it would spend a several hours handling and feeding on the prey then rest for several more hours [133, 149]. Due to the high energetic cost of predation attempts, bears may also rest following unsuccessful hunts [15, 61]. Therefore, the transitions from ARS to stationary/drift states also aligned with our predictions. We did not have any *a priori* predictions on the most likely transition from drift, however the marked higher probability of transitioning to ARS compared to olfactory search was notable. Assuming seals exhibit a patchy distribution, once bears identify a productive patch through olfactory search, they may remain in more localized behaviors (i.e., drift and ARS) as they switch between localized search, hunting, feeding, and resting [133, 149].

For the first 15 h of the day, drift was the most prevalent state with a peak around 08:30 h. Similarly, Ware et al. [161] detected minimal activity before 08:00 h during January–March for the Southern Beaufort Sea polar bear population. Moreover, Stirling [138] identified the first 8 h of the day as the most advantageous period for still-hunting seals, the favored hunting strategy of polar bears during the non-pupping season. Seal haul-out varies diurnally and seasonally. During early winter, seals haul-out primarily at night, while during spring, haul-out behavior peaks around 12:30 h. [7, 46, 73, 138, 158, 159]. Therefore, under the hypothesis that polar bears still-hunt more when seals spend more time in the water, bears should still-hunt during the day during winter, and during spring, bears should still-hunt during the night. However, opposite to this hypothesis Ware et al. [161] found a peak in polar bear activity from 12:00 to 14:00 h during early winter, when seals haul-out at night, and a peak in bear activity at 24:00 h during the pupping season (i.e., April and May), when seals haul-out primarily during the day. The peak in activity we observed in January–March was 8 h later than Ware et al. [161], however in line with Ware et al. [161] we observed a night-time peak in activity in March–May followed by a highly variable timing of activity in June (Additional file 1: Fig. D1). The discrepancies in sleep and activity among studies may be due to population variability, interactions with season, or methodological differences (e.g., inability to differentiate between sleep and still-hunting).

As winter progressed, we observed a decrease in the low energy states (i.e., drift and ARS) and an increase in the higher energy state (i.e., olfactory search). The shift from low energy behaviors to high energy behaviors coincides with seals accessibility and may reflect corresponding changes in ideal hunting strategy from ambush predation to stalking predation [97, 158, 161]. Polar bears exhibit various hunting strategies with corresponding variation in movement and activity [161]. During the first half of winter, seals spend the majority of their time in the water and rarely haul-out to the surface [73, 159]. Thus, access to ringed seals is primarily limited to breathing holes using still-hunting [138]. In April–May, ringed seals give birth and nurse their pups in subnivean lairs as the pups lack the thermal insulation to withstand the cold temperatures of Arctic waters and environment [134]. The subnivean lairs are visually inconspicuous, and polar bears rely on their sense of smell to locate them [142, 152]. Following the pupping season, from late May until the sea-ice melts, seals spend the majority of their time basking on the sea-ice surface to molt [7, 53, 73, 159]. In addition, as the sea ice begins to thaw from mid-May, ringed seals no longer need to rely on breathing holes to surface, reducing the effectiveness of still-hunting [97, 143]. Access to seals is greatest during the pupping and molting season, which corresponds to the peak polar bear foraging period when bears enter hyperphagia [97, 113, 125, 140].

The increase in olfactory search during a period of increased prey availability aligns with the predictions of the marginal value theorem. The theorem states that a predator should exploit a patch until the energy intake rate drops to the average of the entire habitat, which would occur earlier during periods and regions with greater resource availability [21]. It is noteworthy that the marginal value theorem does not necessarily predict the specific mechanism that governs residency, but rather the departure time from a patch [21]. Residency time is an emergent property of discrete behaviors and finer-scale space use. For example, higher residency time can be produced by slower manifestation of some behavior (e.g., slower travel), longer time spent in slower behaviors (e.g., sleeping or nursing), or engaging in slow behaviors associated with patch use (e.g., ARS or feeding). We argue that a behavioral switch from still-hunting to active hunting is the behavioral mechanism that would lead to resource-linked residency time. A behavioral switch can also be viewed in terms of optimal foraging theory. For example, during periods of greater resource availability, animals can afford to engage in more energy-costly behaviors to maximize energy intake, and during periods of reduced resource availability, animals should switch to energy-conserving behaviors [119]. For example, during the summer fasting period when polar bears remain onshore, they exhibit the lowest annual activity in order to minimize energy expenditure (i.e., when compared to the winter foraging period; [10, 107, 109, 152, 162]). In contrast, bears with access to subsistence-harvested whales during the summer exhibit higher activity [160].

Most other polar bear populations exhibit a similar increase in activity coinciding with the seal pupping period [3, 10, 97, 133, 140, 161]. However, some contrasting patterns have been documented in the literature, which may be due to geographic or methodological differences. In areas of land-fast ice, polar bears increase frequency of still-hunting from spring to summer as warmer temperatures expose snow-covered breathing holes and promote still-hunting [142]. In areas of pack ice, warm temperatures promote the formation of open leads and impede still-hunting [143]. Indeed, polar bear movement rates are higher over active ice than over consolidated ice [10, 41]. Parks et al. [109] showed higher movement rates in winter compared to break-up in Hudson Bay, however they did not correct for sea ice motion, which is faster in winter [166] and can artificially inflate movement rates [3, 5].

The decrease in stationary behavior we observed in spring also coincides with the peak in polar bear breeding activity in March–May [41, 148, 161]. Polar bear breeding pairs are associated with reduced time walking and hunting compared to non-breeding pairs resulting in a corresponding range contraction [148], which we expect to manifest as an increase in ARS-like movement. Assuming a typical weaning period between March and June of 2.25-$-$2.5 year old cubs [122, 142], four (one confirmed) of our 14 bears would have been alone and possibly breeding. We expect these bears to have inflated the frequency of ARS in spring, suggesting the observed increase in olfactory search may be conservative among non-breeding individuals. In addition, due to faster wind speeds in early winter, sea ice motion in Hudson Bay is higher earlier in the season [74, 86, 166], which may inflate movement rates, increase misclassification of drift as ARS or olfactory search, and further underestimate the seasonal decline in drift.

We hypothesized two effects of ice concentration on polar bear foraging. First, lower ice concentrations are more energetically costly to move through, wherein bears may have to travel longer distances to avoid swimming [9, 128], which use significantly more energy than walking [33, 52]. Second, ice concentration may influence the distribution and accessibility of seals. As described earlier, high ice concentrations may be more amenable to still-hunting as seal access to surface is more constrained and predictable, while at low ice concentrations, seal access to open water is greater and may promote active search and stalking hunt among polar bears [97, 143]. We observed an increase in the drift state as ice concentration increased in support of both the aforementioned hypotheses. Increased drift state in high ice concentration area is also in line with previous work showing that polar bears select for areas of high ice concentration, where they exhibit greater residency times, and lower movement rate in most studied populations [11, 32, 41, 79, 85, 114]. Some studies identified an unexplained increase in activity with ice concentration [10, 160], contrasting results that may be due to limited location data in low ice concentration in our research, different periods examined, or geographic variation.

There are open leads that encircle Hudson Bay and areas closer to shore tend to be more biologically productive [58, 141], resulting in ringed seals being more likely to remain in a resident behavior in shallower areas close to shore [88]. The open lead is also the primary habitat for harbor seals (*Phoca vitulina*; [6]). Polar bear habitat selection with respect to distance from shore appears to depend on scale. At large scales (e.g., $> 150$ km), polar bears throughout the Arctic appear to select areas closer to shore [32, 68, 79, 92]. However, at a finer scale (e.g., $< 150$ km), polar bears appear to select for habitat further from shore, as the land-fast ice near shore may be less productive and preferentially used by subordinate individuals [68, 114]. In line with previous research, we observed a peak in the probability of being in the stationary drift state around 80 km, with olfactory search being more common closer to shore, and ARS increasing with distances $> 80$ km from shore. Thus, we suggest that there may be an optimal distance to shore rather than a simple monotonic increase or decrease as suggested by previous research. The selected distance to shore is likely specific to population, season, and demographic group. For example, in areas with a narrow continental shelf, such as the Beaufort and Greenland seas, bears may remain closer to shore [32, 68, 79] compared to areas with a broad continental shelf (e.g., Hudson Bay and Laptev and Kara Seas; [32, 91, 92]). In areas with seasonal ice, bears may select areas close to shore to maintain proximity to summering grounds, which is supported by an increasing selection close to shore in break-up compared to winter [92]. Competitive exclusion may also force subordinate individuals (e.g., females with cubs of the year) into lower quality habitat [68, 91, 114].

We predicted olfactory search would be most common at moderate wind speeds since still air and fast winds can impede olfaction [2, 51, 152], and fast or cold winds may encourage polar bears to shelter in place [57] and deter seals from hauling out [20, 46, 53]. However, as wind speed increased, we observed a decrease in drift and increase in ARS. This unexpected relationship between drift and ARS with respect to wind was likely an artifact of our ice motion correction model, which was trained on lower resolution dropped collars. Due to temporal averaging, the model may underestimate sea ice motion caused by wind, causing stationary behavior to be misclassified as ARS at high winds. There is a need for movement models that simultaneously predict and correct for sea ice motion and carry error in drift estimation forward into state classification.

### State distribution

We observed variation in distribution associated with season, mean ice concentration, and bear age. Data from early winter were further from the summer refugia and had a larger range than data from late winter. Studies have found selection for areas closer to shore was stronger in late winter compared to early winter [81, 92, 109]. In contrast to our findings, Durner et al. [35] identified a range expansion from winter into break-up in the Beaufort Sea that appeared to reflect a regime shift from stable multi-year sea ice to seasonal ice resulting in some of bears migrating south in the summer and some to range north following the retreating sea ice [108, 115].

The bear utilization distribution was greater in years with low ice concentration, which may be a response to habitat fragmentation that encourages bears to range further in search of quality habitat. Only $28\%$ of the data points occurred in years with low ice concentration, suggesting that the observed area of the UD may be a conservative estimate. A similar negative relationship with sea ice concentration and home range size was found in several studies for the Southern Beaufort Sea population [3, 35, 40, 56, 108]. However, other work showed home range to increase with an extended ice season and greater extent [81, 109]. One possible explanation for the differences among studies may be that some populations experience habitat fragmentation (including Western Hudson Bay) while others experience habitat loss [12, 39]; habitat fragmentation may increase ranging in search of quality patches (e.g., [49, 89, 108]), while habitat loss may lead to declines in extent as parts of the historic range become unavailable (e.g., [60, 137]). If this hypothesis is correct, we predict that early stages of sea-ice decline (seasonal or interannual) may promote home range expansion, while latter stages of decline may cause home range decline. It may also be possible for a population to experience both habitat loss and fragmentation simultaneously or depending on season (e.g., increased fragmentation in winter, but habitat loss in spring). Since the effects of habitat fragmentation or loss on home range size are opposite, the change in home range likely depends on the magnitude of loss and fragmentation.

Lastly, we observed that the utilization distribution of younger bears (6–14 years) extended further from the summer refugia than older bears (15–20 years). One possible explanation is intraspecific competition, wherein older, dominant individuals may force subordinates into less optimal habitat through competitive exclusion or kleptoparasitism, a pattern observed between dominant males and solitary females relative to subordinate subadults and females with cubs in other polar bear populations [45, 68, 78, 91, 114, 138, 149]. In the Baffin Bay and East Greenland populations, the coarse-scale (4-day) movement of male polar bears is more tortuous and localized than females, likely to remain in high quality habitat and decrease encounter rates with other males while maintaining similar encounter rates with females, whose movement is more consistent with prey localization [78]. Alternatively, older females with cubs may be more experienced in avoiding males than younger mothers, and therefore able to remain in higher quality habitat, while inexperienced mothers may move further for additional safety from kleptoparasitism [91]. However, little is known about the fine-scale movement and space use of male bears as they cannot be collared. An alternative explanation is age-specific navigational effectiveness, wherein younger bears have poorer navigation abilities on the dynamic drifting sea ice and move further from the summer refugia. Similarly, migrating passerines exhibit a “coastal effect” where younger, inexperienced passerines stray further from the optimal migration flyway compared to adults [121]. The lower navigation abilities hypothesis is supported in our observed behavior probability, which revealed that older individuals spent more time in low energy states. The marginal value theorem predicts that residency time should increase with patch quality, suggesting that older individuals may be more effective at locating higher quality patches [21].

Our sample size ($n=14$) was low and exclusively females, therefore, our results likely do not reflect the diversity of behavioral strategies among polar bears (e.g., [91, 114]). We incorporated orientation bias relative to wind to help differentiate behaviors with similar movement characteristics. This approach is limited when the external factor is spatiotemporally autocorrelated, as only the first few steps in a taxic behavior may display orientation bias, and once the desired angle is obtained, movement appears autocorrelated [8, 26, 155]. Behaviors of similar, or shorter, duration than the telemetry data or behavior transitions that occur between location data may lead to misclassification. In addition, low resolution environmental data could hinder the classification of states with emission probabilities dependent on environmental covariates. For example, even with high resolution telemetry data, we may misclassify up-wind olfactory scent localization if the wind data was of insufficient resolution. Future work should aim to validate state classification. For example, Pagano et al. [104] utilized GPS collars equipped with video cameras to validate accelerometer-derived behavioral states. In addition to state interpretation, it is not self-evident which behaviors are the most ecologically important. For example, olfactory search may represent optimal search strategy when conditions are favorable or a longer search for higher quality habitat [64]. However, the diurnal presence of all three states throughout winter suggest that all three play an important ecological role. Investigating behaviors across seasons with resource variability may provide additional contrast to identify behavioral signatures associated with quality habitat [23, 107, 152, 160, 162]. In addition, future research should consider interactions between covariates; for example, previous research revealed seasonally varying diurnal movement and activity patterns, which may be better modeled with an interaction [109, 159, 161]. Last, as analytical techniques continue to advance, programming at least some transmitters to a higher location frequency (e.g., 1 h or 30 min) would enable research of finer-scale behaviors. Identifying baselines for behavioral time budgets may be increasingly important as environmental conditions continue to change.

### Conclusion

Different behaviors have unique fitness and ecological consequences. Quantifying behavioral time-budgets and factors that promote different behaviors is key to understanding a species’ ecology. Remote tracking has elucidated much about polar bear habitat use [77], however behavioral research has typically been limited to two states or less (e.g., [104, 107], but see [4, 161]). Using advanced models that integrate wind data [155], our study described previously undocumented circadian patterns in Western Hudson Bay polar bears, as well as behavioral variation with respect to season and ice concentration that appear to reflect a shift in foraging strategy in response to a change in prey availability (i.e., increase in haul-out behavior from early winter to the spring pupping and molting seasons). Last, we identified spatial patterns of distribution with respect to season, ice concentration, and bear age that may be indicative of habitat quality and competitive exclusion. Our findings expand on phenologic variation among polar bear populations that may be associated with regional or temporal variation in resource abundance or distribution. Due to the high degree of variation in ice dynamics throughout the Arctic, it is difficult to draw conclusions across populations [3, 74, 78]. Given the circumpolar distribution of polar bears, each population is experiencing a different level of climate change related effects, stressing the need for population-specific research. The focal population of this paper—the Western Hudson Bay—is near the southern limits of the species’ range and is among the most affected by climate change. Our findings stress the importance of accounting for both behavior and the temporal and environmental factors that affect behavior. Failing to account for temporal factors that affect space use may obscure important habitat associations. For example, as polar bear behavior changes seasonally, resource selection functions that do not account for season may miss important patterns of habitat selection. Therefore, our methodology can help identify periods, locations, and environmental conditions that are associated with habitat quality to can help to better understand polar bear behavioral ecology and aid conservation.

**Table 1:** Description of notation used in this paper and their interval, if applicable

Variable	Interval	Description
*T*	$\{1,2,\ldots \}$	Total number of time steps
*t*	[1, *T*]	A time step
$X_t$	–	Set of observations at time *t*
${{\textbf {X}}}$	–	The set of all observations $(X_1,\ldots , X_T)$
*l*	$(0,\infty )$	Step length between consecutive locations
$\phi$	$(-\pi ,\pi ]$	Turning angle (i.e., change in bearing) between consecutive steps
*r*	$[0, \infty )$	Magnitude of the stimulus
$\psi$	$(-\pi ,\pi ]$	Direction of a stimulus relative to the bearing of the previous time step
$\mu ^{(l)}$	$(0,\infty )$	Mean parameter of step length
$\sigma ^{(l)}$	$(0,\infty )$	Standard deviation parameter of step length
$\beta _1$	$(-\infty , \infty )$	Intercept coefficient for mean step length
$\beta _2$	$(-\infty , \infty )$	Slope coefficient for mean step length and *r*
$\mu ^{(\phi )}$	$(-\pi ,\pi ]$	Mean parameter of turning angle
$\kappa ^{(\phi )}$	$(0,\infty )$	Concentration parameter of turning angle
$\alpha _1$	$(-\infty , \infty )$	Bias coefficient in the same direction as the stimulus
$\alpha _2$	$(-\infty , \infty )$	Bias coefficient $90^{\circ }$ left of the stimulus
$\vartheta$	$(-\pi ,\pi ]$	The direction of bias relative to stimulus
$M^*$	[0, 1)	Scaled magnitude of attraction
$\hat{}$	–	An estimated value, e.g., ${\hat{\mu }}^{(l)}$ is the estimated value of $\mu ^{(l)}$
*N*	$\{1,2,\ldots \}$	Total number of behavioral states
*S*	[1, *N*]	Behavioral state
$\varvec{S'}$	[1, *N*]	Behavioral state with the highest within-cell proportion relative to across-cell proportion
$\gamma _{i,j}$	[0, 1]	Transition probability from state *i* to state *j*
$\mathbf {\Gamma }$	–	$N \times N$ Transition probability matrix
*D*	–	The drift state
$O^{(L)}, O^{(R)}$	–	Olfactory search state with anemotaxis left of wind and anemotaxis right of wind, respectively
*ARS*	–	Area-restricted search state

**Table 2:** Parameter estimates for four-state HMM

State	${\hat{\mu }}^{(l)}$[$\dag$]	${\hat{\sigma }}^{(l)}$[$\ddag$]	${\hat{\vartheta }}$[$\S$]	${\hat{\kappa }}^{(\phi )}$[$\P$]	${\hat{M}}^*$[$\Vert$]
Drift, *D*	0.63	0.31	NA	3.64	0.41
Area-restricted search, *ARS*	0.76	0.59	NA	0.62	NA
Olfactory search, $O^{(L, R)}$	2.04	0.93	92	4.28	0.29

[$^\dag$]Mean step length (km h$^{-1}$)

[$^\ddag$]Step length standard deviation (km h$^{-1}$)

[$^\S$]Angle of attraction relative to wind $(^{\circ })$

[$\P$]Turning angle concentration

[$^\Vert$]Scaled magnitude of attraction

## Supplementary Information

**Additional file 1**. Contains supplementary figures, tables, methods, and results. The file contains hypotheses and predictions associated with each covariate used in modelling (Table A1), description of all data sources used in analysis (Table A2), methods and results for t correction and tidal integration (Section B), methods and results for satellite-ice-drift-based sea ice drift correction (Section C), methods and results of HMM without drift correction (Section D.1), HMM using wind and tidal integration (Section D.2), HMM using satellite-based drift correction (Section D.3), and HMM using wind-based drift correction and tidal integration (Section D.4), comparison of different drift correction and integration approaches and discussion of results (Sections D.5 and D.6), structure of pseudo-design matrices used to formulate HMM emission probabilities (Section E), effect of hour and month on state frequency (Figure F1), and distribution and frequency of predicted states in Hudson Bay (Figure G1).

## Data Availability

The data-set supporting the conclusions of this article is available in the University of Alberta Dataverse repository, [DOI and hyperlink will be made available upon acceptance].

## Abbreviations

aKDE: Autocorrelated kernel density estimator
BRW: Biased random walk
CRW: Correlated random walk
BCRW: Biased correlated random walk
HMM: Hidden Markov model
UD: Utilization distribution
