# Supplementary material for: Drivers of polar bear behavior and the possible effects of prey availability on foraging strategy
Source: Mov Ecol. 2022 Nov 16;10:50. doi: 10.1186/s40462-022-00351-4 (PMC9670556; doi:10.1186/s40462-022-00351-4)
Supplement: Supplementary file 1 — Additional file 1. Contains supplementary figures, tables, methods, and results. The file contains hypotheses and predictions associated with each covariate used in modelling (Table A1), description of all data sources used in analysis (Table A2), methods and results for t correction and tidal integration (Section B), methods and results for satellite-ice-drift-based sea ice drift correction (Section C), methods and results of HMM without drift correction (Section D.1), HMM using wind and tidal integration (Section D.2), HMM using satellite-based drift correction (Section D.3), and HMM using wind-based drift correction and tidal integration (Section D.4), comparison of different drift correction and integration approaches and discussion of results (Sections D.5 and D.6), structure of pseudo-design matrices used to formulate HMM emission probabilities (Section E), effect of hour and month on state frequency (Figure F1), and distribution and frequency of predicted states in Hudson Bay (Figure G1). [file 40462_2022_351_MOESM1_ESM.pdf]

A Covariate hypotheses, predictions, and data sources

Table A1: Hypotheses and predictions associated with each behavioral state (drift, area restricted search (ARS), and olfactory search). Cells with the same content as the previous row are indicated by the quotation mark.

Covariate	Hypotheses	Prediction
Ordinal date	- Access to prey is linked to seasonal variation in seal abundance, distribution, and behavior<br>- Seal abundance is highest during the pupping season in spring<br>- Seal basking and haul-out behavior is highest during spring molting season	As the season progresses, olfactory search and ARS states will increase and the drift state will decrease
Local hour	- Behavior exhibits circadian patterns associated with resource availability (i.e., seal distribution and behavior)<br>- Access to seals is greatest during mid-day when haul-out behavior is most frequent<br>- Low ambient light increases the reliance on olfaction	The drift state will peak 0:00 h, olfactory search will be most common in the early morning and late evening, and ARS will be most common during the day
Solar radiation	“	“
Sun altitude	“	“
Wind velocity	- Low wind speed do not facilitate scent dispersion and hinder olfaction<br>- High wind speed increases turbulence, fragments odor plumes, and hinders olfaction<br>- High wind speed decrease seal haul-out behavior and decrease access to prey	The drift state will be most common during low and high wind speeds and olfactory search and ARS will peak at moderate speeds
Snow depth	- Increasing snow depth increases the energetic cost of locomotion and decreases the effectiveness of olfaction	Olfactory search will be negatively correlated with snow depth, and drift and ARS states will be positively correlated with snow depth
Total precipitation	- Precipitation hinders olfactory and visual search	Olfactory search will be negatively correlated with snow depth, and drift state will be positively correlated with snow depth<br>ARS may be negatively correlated or peak at intermediate levels of precipitation
Sea ice concentration	- Low ice concentration increases cost of locomotion.<br>- Low ice concentration decreases accessibility of prey<br>- Still-hunting is most favorable at high ice concentration when seal access to air is more limited	Olfactory search and drift will be positively correlated with ice concentration, and ARS will be negatively correlated with ice concentration.
Bathymetry	- Land-fast ice over shallow waters is unproductive and has low resource availability	Areas further from shore (i.e., deeper waters) will promote more localized behaviors (i.e., ARS and drift)

Table A2: Description of data sources used in this paper, their spatial and temporal resolutions (res.), and the method used to interpolate data at polar bear locations.

Covariate	Data product	Data producer	Data source	Spatial res.	Temporal res.	Interpolation
Ordinal date	GPS collar	Telonics	NA	NA	NA	NA
Local hour	GPS collar	Telonics	NA	NA	NA	NA
10 m wind velocity	ERA5 meteorological reanalysis project	ECMWF	[Hersbach et al., 2020]	0.25° (∼21 km)	1 h	Bilinear
Snow depth	ERA5 meteorological reanalysis project	ECMWF	[Hersbach et al., 2020]	0.25° (∼21 km)	1 h	Bilinear
Total precipitation	ERA5 meteorological reanalysis project	ECMWF	[Hersbach et al., 2020]	0.25° (∼21 km)	1 h	Bilinear
Surface solar radiation	ERA5 meteorological reanalysis project	ECMWF	[Hersbach et al., 2020]	0.25° (∼21 km)	1 h	Bilinear
Sun altitude	Sun altitude	British Geological Survey	[Kelley et al., 2022]	1 m	Instantaneous	NA
Sea ice concentration	Bootstrap Sea Ice Concentrations from Nimbus-7 SMMR and DMSP SSM/ I-SSMIS, Version 2	NASA	[Comiso, 2017]	25 km	24 h	Trilinear
Bathymetry	General Bathymetric Chart of the Oceans	IHO & IOC	[IOC et al., 2003]	1/240° (∼350 m)	NA	Extract
Tidal currents	Tidally-driven ice drift speed due to M2 tide	Institut des Sciences de la Mer, Universitè du Quèbec à Rimouski	[St-Laurent et al., 2008]	10 km	Instantaneous	Bilinear
Sea ice motion vectors	Polar Pathfinder Daily 25 km EASE-Grid Sea Ice Motion Vectors, Version 4	NASA	[Tschudi et al., 2019, 2020]	25 km	24 h	Trilinear

Acronyms: ECMWF, European Centre for Medium-Range Weather Forecasts; ERA5, ECMWF reanalysis version 5; GPS, global positioning system; IHO, International Hydrographic Organization; IOC, Intergovernmental Oceanographic Commission; NA, not applicable; NASA, The National Aeronautics and Space Administration.

B Drift correction

B.1 Drift correction methods

Togunov et al. [2020] identified 20 drifting collars with locations within Hudson Bay between 2005–2015 that yielded 10,409 locations during the months of December–July. To remove data-sparse periods, we segmented the tracks at any gaps larger than 24 hours and discarded segments with fewer than 16 locations. To obtain a regular time series for each track segment, we imputed missing locations by fitting a continuous-time correlated random walk model using the `crawl` package in `R` [Johnson and London, 2018, Johnson et al., 2008, RStudio Team, 2021]. After filtering sparse locations and imputing missing locations (n = 725; 6.79%) each collar had a mean of 562 ± 347 GPS fixes (total = 10675).

Sea ice drift is driven by wind forcing, ocean currents, the Coriolis force, and internal ice stress. Due to the compressive strength of sea ice, local drift is typically affected by average force applied to it at a large scale. To allow for this, we summarized wind velocities at four buffers (0 km, 50 km, 100 km, 150 km). The buffer represents the radius around each GPS location from which wind velocities were extracted. The wind velocities of all raster cells that fell within the buffer were averaged to obtain the an estimate for the GPS point. For the buffer of 0 km, wind speed was estimated at the GPS location using bilinear interpolation. The buffer with the lowest AIC was 100 km and was used to predict drift. Wind velocities were obtained from the ERA5 meteorological reanalysis project, which provides hourly global analysis fields at a 31 km resolution [Hersbach et al., 2020] and were extracted using the `raster` package [Hijmans and van Etten, 2016].

The motion of the GPS tracks was described using two data streams: step length $l_t \in (0, \infty)$ (the distance between consecutive locations) and turning angle $\phi_t \in (-\pi, \pi]$ (change in bearing between consecutive steps), where $t \in 1, ..., T$ represents the time of the first GPS location in a step [Langrock et al., 2012, McClintock and Michelot, 2018]. Step length l_t and turning angle ϕ_t were modelled as slope-intercept functions of wind velocities. We fit Weibull and gamma distributions to step length and von Mises and wrapped Cauchy distributions to turning angle and selected the combination of distributions that had the lowest AIC [McClintock et al., 2020]. Our final model for drift assumed that step length follows a Weibull distribution:

$$l_t \sim \text{Weibull}(\kappa_t^{(l)}, \lambda_t^{(l)}), \tag{1}$$

where $\kappa_t^{(l)} > 0$ is the shape parameter and $\lambda_t^{(l)} > 0$ is the scale parameter of the step length [McClintock et al., 2020]. The shape and scale parameters were each defined as slope-intercept functions of wind speed

$r_t \geq 0$ as follows:

$$\ln(\kappa_t^{(l)}) = \beta_1 + \beta_2 r_t \tag{2}$$

$$\ln(\lambda_t^{(l)}) = \beta_3 + \beta_4 r_t, \tag{3}$$

where is $\beta_{\{1,2,3,4\}} \in \mathbb{R}$ are the intercept and slope coefficients to be estimated.

According to AIC, our best model defined turning angle ϕ_t using the von Mises distribution. Due to the Coriolis effect, sea ice drift is approximately -20°relative to the wind direction in the northern hemisphere [Tschudi et al., 2010]. However, the precise angle depends on a number of factors and was estimated closer to -15°in Hudson Bay [Togunov et al., 2021]. To allow for drift toward an unknown angle of bias relative to wind, we modelled the mean turning angle as a trade-off between bias parallel to the direction of the wind and bias perpendicular to wind. Specifically, mean turning angle $\mu_t^{(\phi)}$ was assumed to follow a menotactic BRW circular-circular von Mises regression model [Rivest and Duchesne, 2016, Togunov et al., 2021]:

$$\begin{aligned}\mu_t^{(\phi)} = \operatorname{atan2}(&\alpha_1 \sin\psi_t + \alpha_2 \cos\psi_t,\\ &\alpha_1 \cos\psi_t - \alpha_2 \sin\psi_t),\end{aligned} \tag{4}$$

where ψ_t is the direction of wind at t relative to the movement bearing at $t-1$. We hypothesized that the concentration of turning angle around $\mu_t^{(\phi)}$ would increase as wind speeds increased. At higher wind speeds, the force is greater and the direction can be predicted more accurately. To capture this, we modeled turning angle concentration as a slope-intercept function of wind speed following:

$$\ln(\kappa_t^{(\phi)}) = \beta_5 + \beta_6 r_t. \tag{5}$$

After fitting the BCRW drift model to the dropped collar data, we predicted the drift speed and direction given the estimated ECMWF wind vectors. Shape and scale parameters of step length were estimated using Eq. 2 and Eq. 3, which were used to estimate the latent drift speed $\hat{l}_t$ as the mode of Weibull step length following:

$$\hat{l}_t = \begin{cases} 0 & \text{if } \hat{\kappa}_t^{(l)} \leq 1 \\ \hat{\lambda}_t \left(\dfrac{\hat{\kappa}_t^{(l)} - 1}{\hat{\kappa}_t^{(l)}}\right)^{1/\hat{\kappa}_t^{(l)}} & \text{if } \hat{\kappa}_t^{(l)} > 1. \end{cases} \tag{6}$$

We used the estimated mode drift speed for the correction as it minimized the median drift-corrected step length $l_t^{(c)}$ compared to the mean or median of the Weibull distribution.

Mean direction $\hat{\mu}_t^{(\phi)}$ of drift was predicted using Eq. 4. Next, we estimated the drift-corrected paths by subtracting the estimated drift vectors from the observed drift vectors following:

$$\begin{aligned} \hat{u}_t &= \hat{l}_t \cos \hat{\mu}_t^{(\phi)} \\ \hat{v}_t &= \hat{l}_t \sin \hat{\mu}_t^{(\phi)}, \end{aligned} \tag{7}$$

where $\hat{u}_t \in \mathbb{R}$ and $\hat{v}_t \in \mathbb{R}$ are the estimated east-to-west and south-to-north components of the drift-corrected track. The estimated drift-corrected step length $\hat{l}_t^{(c)}$ was calculated following:

$$\hat{l}_t^{(c)} = \sqrt{\hat{u}_t^2 + \hat{v}_t^2}. \tag{8}$$

Next, we estimated the drift-corrected trajectory of the track using the cumulative sum of the estimated u_t and v_t components following:

$$\begin{aligned} \hat{x}_t^{(c)} &= \begin{cases} [h]0 & \text{if } t = 1 \\ \sum_{i=1}^{t-1} \hat{u}_i & \text{Otherwise} \end{cases} \\ \hat{y}_t^{(c)} &= \begin{cases} 0 & \text{if } t = 1 \\ \sum_{i=1}^{t-1} \hat{v}_i & \text{Otherwise.} \end{cases} \end{aligned} \tag{9}$$

where $\hat{x}_t^{(c)}$ and $\hat{y}_t^{(c)}$ are the estimated x and y coordinates of the drift-corrected track, respectively.

To test that we did not over-fit the data, we evaluated the drift correction BCRW performance in reducing the apparent speed of dropped collars by fitting it to a random subset of 10% of the dropped collar tracks and correcting for it in the entire dropped collar data set. When correcting for drift in the bear tracks, we fit the drift correction BCRW to the entire dropped collar data-set, and used the estimated coefficients and ECMWF wind to predict and correct for drift along the bear tracks.

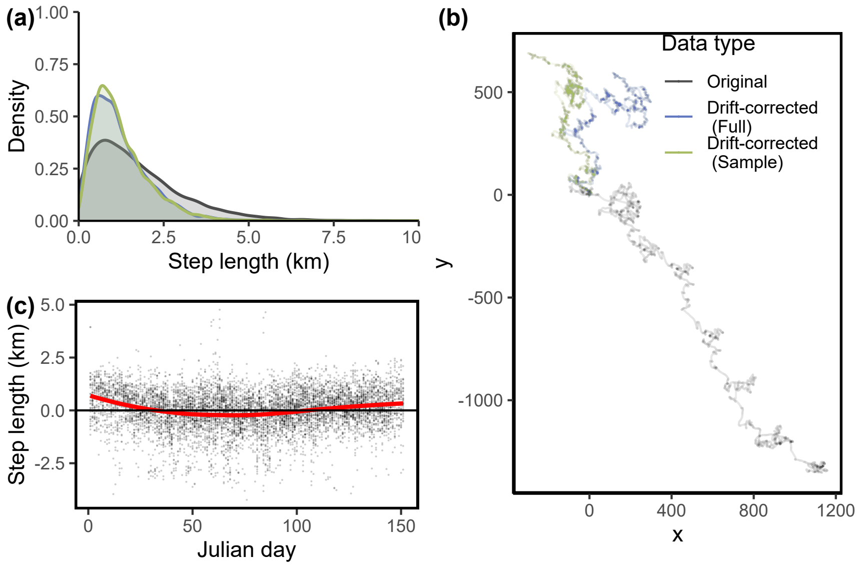

Figure B1: Drift correction: (a) Step length of dropped collars before and after drift correction; (b) cumulative path of dropped collars before and after drift correction; (c) residual plot of step length for drift corrected dropped collars sorted by ordinal date. Grey represents original data, blue represents drift-corrected data based on BCRW fit to the full data set, and green represents drift-corrected data based on BCRW fit to a 10% random sample of dropped collar tracks.

B.2 Drift correction results

We expect that applying the drift correction would mostly reduce the movement of the drop collar close to zero. When fit to the full dropped collar data set, the drift correction reduced the mean step length by 29% from 1.76 km to 1.25 km (Fig B1). When fit to a 10% random sample of dropped collar tracks, the drift correction reduced the mean step length by 28% to 1.26 km. (Fig. B1). The drift corrected tracks still appear to have a remaining directional bias, however it is less than the original track (Fig. B1). There appears to be some residuals with respect to day of the year (Fig. B1C), however, including day of the year in the drift correction BCRW yielded only marginal model improvement with respect to reduction of step length (data not shown).

C Ice motion-based drift correction

C.1 Drift correction methods

We tested using sea ice motion vectors produced by the National Snow and Ice Data Center [NSIDC; Tschudi et al., 2019, 2020] to correct for ice motion in the dropped collars as in Appendix B.

All the same procedures as described in Appendix B.1 were followed with the exception of the definition of step length $l_t \in (0, \infty)$ and turning angle $\phi_t \in (-\pi, \pi]$ being defined in terms of sea ice drift rather than wind velocities. Specifically, the shape and scale parameters of step length were defined as slope-intercept functions of NSIDC ice motion speed $r_t^{(NSIDC)} \geq 0$ as follows:

$$\ln(\kappa_t^{(l)}) = \beta_1 + \beta_2 r_t^{(NSIDC)} \tag{10}$$

$$\ln(\lambda_t^{(l)}) = \beta_3 + \beta_4 r_t^{(NSIDC)}, \tag{11}$$

where is $\beta_{\{1,2,3,4\}} \in \mathbb{R}$ are the intercept and slope coefficients to be estimated.

Mean turning angle $\mu_t^{(\phi)}$ was assumed to be a BRW circular-circular von Mises regression model [Rivest and Duchesne, 2016]:

$$\begin{aligned} \mu_t^{(\phi)} = \operatorname{atan2}(&\alpha_1 \sin \psi_t^{(NSIDC)}, \\ &\alpha_1 \cos \psi_t^{(NSIDC)}), \end{aligned} \tag{12}$$

where $\psi_t^{(NSIDC)}$ is the direction of NSIDC ice motion at t relative to the movement bearing at $t-1$. In addition, we modeled turning angle concentration as a slope-intercept function of NSIDC ice motion speed following:

$$\ln(\kappa_t^{(\phi)}) = \beta_5 + \beta_6 r_t^{(NSIDC)}. \tag{13}$$

After fitting the BCRW drift model to the dropped collar data, we predicted the drift speed and direction given the estimated NSIDC ice motion vectors. Shape and scale parameters of step length were estimated using Eq. 10 and Eq. 11 and were used to estimate the latent drift speed $\hat{l}_t$ using eq. 6. Mean drift direction $\hat{\mu}_t^{(\phi)}$ of drift was predicted using Eq. 12. Finally, we estimated the drift-corrected paths, step lengths, and trajectory as described in Appendix B.1 using eq. 7, eq. 8, and eq. 9, respectively

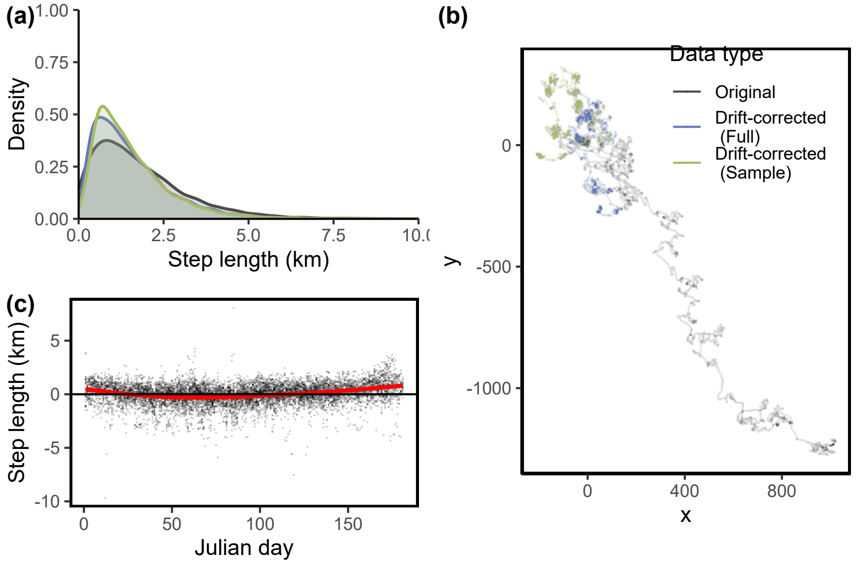

Figure C1: Drift correction: (a) Step length of dropped collars before and after drift correction using NSIDC sea ice motion vectors; (b) cumulative path of dropped collars before and after drift correction; (c) residual plot of step length for drift corrected dropped collars sorted by ordinal date. Grey represents original data, blue represents drift-corrected data based on BCRW fit to the full data set, and green represents drift-corrected data based on BCRW fit to a 10% random sample of dropped collar tracks.

C.2 Drift correction results

When fit to the full dropped collar data set, the drift correction reduced the mean step length by 14% from 1.76 km to 1.51 km; Fig. C1). When fit to a 10% random sample of dropped collar tracks, the drift correction reduced the mean step length by 14% from 1.76 km to 1.51 km (Fig. C1).

D Drift correction HMM output comparison

This subsection compares broad differences in HMM output for four different methods of accounting for ice drift in telemetry data: 1) no drift correction/integration, 2) wind-based drift and tidal integration, 3) satellite-based drift vector subtraction and tidal integration, and 4) wind-based BCRW vector subtraction and tidal integration. The first method assumes there is no effect of sea ice drift on bear movement and only the emission probabilities of turning angle for olfactory search are modeled as a function of wind. The second method integrates both wind and tidal drift directly into the emission probabilities for step length and turning angle. The third method implements the classical approach of subtracting satellite-based estimating drift (NSIDC's 'Polar Pathfinder Daily 25 km EASE-Grid Sea Ice Motion Vectors, Version 4') as well as tidal circulation. The final method, is the one described in Appendix B, where ice drift is estimating using a BCRW model of wind-driven drift fit to dropped collars, then predicted/subtracted from the polar bear data, and the remnant tidal circulation is modeled.

D.1 No ice motion correction or integration

The first method assumes ice motion has no effect on bear movement. Step length is modeled as intercept-only for each state (i.e., main text, equation 1). Turning angle is modeled following:

$$\mu^{(\phi)}_{S,t} = \begin{cases} 0 & \text{if } S = otherwise, \\ \text{atan2}(\alpha_1 \sin\psi_t^{(wind)} - \alpha_2 \cos\psi_t^{(wind)}, & \text{if } S = O^{(R)}, \\ \qquad 1 + \alpha_1 \cos\psi_t^{(wind)} + \alpha_2 \sin\psi_t^{(wind)}) & \\ \text{atan2}(\alpha_1 \sin\psi_t^{(wind)} + \alpha_2 \cos\psi_t^{(wind)}, & \text{if } S = O^{(L)}, \\ \qquad 1 + \alpha_1 \cos\psi_t^{(wind)} - \alpha_2 \sin\psi_t^{(wind)}) & \end{cases} \tag{14}$$

where $\psi_t^{(wind)}$ represents the directions of wind at time t relative to the track bearing at time $t-1$, α_1 represents the magnitude of attraction toward $\psi_t^{(wind)}$ for the olfactory search states, and α_2 represents the magnitude of attraction toward $\psi_t^{(wind)} + \pi/2$ for the olfactory search states [Togunov et al., 2021]. In this formulation, it is assumed that the direction of D and ARS is centered at 0 and that the direction of olfactory search states is exclusively detriment by the direction of wind.

D.2 Wind-based drift and tidal integration

In the second method, step length $\mu^{(l)}_{S,t}$ is modeled as follows:

$$\mu^{(l)}_{S,t} = \begin{cases} \beta_{1,S} + \beta_2 r^{(wind)}_t + \beta_3 r^{(tide)}_t \cos(\theta^{(tide)}_t - \theta^{(wind)}_t + 0.26) & \text{if } S = D \\ \beta_{1,S} + \beta_2 r^{(wind)}_t \cos(\theta^{(track)}_t - \theta^{(wind)}_t + 0.26) & \text{Otherwise,} \end{cases} \tag{15}$$

where $\beta_{1,S}$ is the state-specific intercept coefficient for step length, β_2 is the slope coefficient for the effect of wind speed on drift speed, $r^{(wind)}_t$ is the estimated wind speed at time t, β_3 is the slope coefficient for the effect of tidal circulation on drift speed, $\theta^{(tide)_t}$ is the direction of tidal circulation at time t, $\theta^{(wind)}_t$ is the direction of wind at time t, and $\theta^{(track)}_t$ is the direction of track at time t. Note, in this formulation, it is assumed wind is the primary driver of drift, and only the component of tidal circulation in the same direction as wind-driven drift is added to drift speed $(\cos(\theta^{(tide)}_t - \theta^{(wind)}_t + 0.26)$. 0.26 radians (15°) is added to the difference between $\theta^{(tide)_t}$ and $\theta^{(wind)_t}$ as drift tends to occur 15° right relative to wind. It is also assumed that when the bear is active, tidal drift has a negligible effect on the observed step length and only the component of wind-based drift parallel to the orientation of the bear is added to the step length $(\cos(\theta^{(track)}_t - \theta^{(wind)}_t + 0.26))$. A unique step length standard deviation assumed for each state.

Turning angle is modeled following:

$$\mu^{(\phi)}_{S,t} = \begin{cases} \text{atan2}(\alpha_{1,D} r^{(wind)}_t \sin(\psi^{(wind)}_t - 0.26) + \alpha_{2,D} r^{(tide)}_t \sin\psi^{(tide)}_t, & \text{if } S = D, \\ \qquad 1 + \alpha_1 r^{(wind)}_t \sin(\psi^{(wind)}_t - 0.26) + \alpha_{2,D} r^{(tide)}_t \sin\psi^{(tide)}_t & \\ 0 & \text{if } S = ARS, \\ \text{atan2}(\alpha_{1,O} \sin\psi^{(wind)}_t - \alpha_{2,O} \cos\psi^{(wind)}_t, & \text{if } S = O^{(R)}, \\ \qquad 1 + \alpha_{1,O} \cos\psi^{(wind)}_t + \alpha_{2,O} \sin\psi^{(wind)}_t) & \\ \text{atan2}(\alpha_{1,O} \sin\psi^{(wind)}_t + \alpha_{2,O} \cos\psi^{(wind)}_t, & \text{if } S = O^{(L)}, \\ \qquad 1 + \alpha_{1,O} \cos\psi^{(wind)}_t - \alpha_{2,O} \sin\psi^{(wind)}_t) & \end{cases} \tag{16}$$

where $\psi^{(wind)}_t$ and $\psi^{(tide)}_t$ represent the directions of wind and tides, respectively, at time t relative to the track bearing at time $t-1$, $\alpha_{1,D}$ represents the magnitude of attraction toward $\psi^{(wind)}_t - 0.26$ for the drift state, $\alpha_{2,D}$ represents the magnitude of attraction toward $\psi^{(tide)}_t$ for the drift state, $\alpha_{1,O}$ represents the magnitude of attraction toward $\psi^{(wind)}_t$ for the olfactory search states, and $\alpha_{2,O}$ represents the magnitude of attraction toward $\psi^{(wind)}_t + \pi/2$ for the olfactory search states [Togunov et al., 2021]. In this formulation, it

is assumed that the direction of drift only affects the drifting state, the ARS state turning angle is centered at 0, and that the direction of olfactory search states is exclusively detriment by the direction of wind.

D.3 Satellite-based drift vector subtraction and tidal integration

In the third method, NSIDC sea ice motion vectors (horizontal u and vertical v components) are first interpolated to the track location using tri-linear interpolation (first, bilinearly in space, then linearly in time). Next, the $u^{(NSIDC)}$ and $v^{(NSIDC)}$ components of drift are subtracted from the horizontal and vertical components of track displacement to obtain voluntary displacement fol owing:

$$\begin{aligned} u_t^{(bear)} &= x_t^{(track)} - x_{t-1}^{(track)} - u^{(NSIDC)} \\ v_t^{(bear)} &= y_t^{(track)} - y_{t-1}^{(track)} - v^{(NSIDC)}, \end{aligned} \tag{17}$$

where $u_t^{(bear)}$ and $v_t^{(bear)}$ are the voluntary eastward and northward displacements by the bear, respectively (not accounting for tidal circulation), $x^{(track)}$ and $y^{(track)}$ are the projected in m (e.g., UTM NAD83, EPSG:5321) coordinates obtained by the collars, and $u^{(NSIDC)}$ and $v^{(NSIDC)}$ are the eastward and northward components of NSIDC sea ice motion vectors, respectively in $m/(t_i - t_{i-1})$.

Mean step length is described as in equation 2 in the main text, and mean turn angle is as described in equation 4 in the main text.

D.4 Wind-based BCRW vector subtraction and tidal integration

The fourth method is the primary method described in the main text. First, wind-driven ice drift is estimated and subtracted from the polar bear tracks as described in Appendix A. Then remnant movement is modeled as described in main text equations 1-4.

D.5 Results

Based on the Viterbi-decoded states, the state frequencies were relatively comparable between the integration, satellite-correction, and BCRW-connection models: 17–19% in ARS, 46–51% in stationary drift, and 32–36% in olfactory search (Table D1). The HMM based on the raw data with no ice drift integration or correction exhibited a markedly lower frequency of ARS (12%) and stationary drift (22%), but nearly twice the frequency of olfactory search (66%). This is likely because the added motion of sea ice makes it much more difficult to identify stationary or low-velocity behaviors, while inflating the frequency of behaviors with

directed travel.

When comparing specific decoded states, the concurrency of each model combination varied between 45–85% (Table D2). The largest discrepancies between models was between the raw HMM and the BCRW-based correction, followed by NSIDC-based correction. Among the models with drift correction and integration, concurrency ranged between 78–85% and the largest state discrepancy occurred in the classification of olfactory search. Specifically, when comparing the ice drift integration to the satellite-based correction (69% discovery rate) and BCRW correction (68% discovery rate; Table D2). ARS had the next lowest discovery rate between 80–87% among HMMs with drift correction/integration (Table D2). In every confusion matrix, both ARS and drift are most frequently classified as olfactory search, and olfactory search is classified as drift.

The difference in drift correction between the satellite-based method versus the BCRW-based method is particularly apparent in Fig. D1 (c) versus (d), wherein the segments classified as drift are more contracted in the BCRW-based method. The difference between the estimated step length and turning angle distributions are relatively minor between the different models (Fig. D1 e-l).

Table D1: State frequency (as percentage) for HMMs based on each drift correction model (Raw, no drift correction or integration; Integ., wind-based drift and tidal integration; NSIDCc, satellite-based drift vector subtraction and tidal integration; and BCRWc, wind-based BCRW vector subtraction and tidal integration). The classified states were area restricted search (ARS), drift (D), and olfactory search (O).

	ARS	D	O
Raw	12	22	66
Integ.	18	46	36
NSIDCc	19	47	34
BCRWc	17	51	32

D.6 Conclusions

The most confidently identifiable state is stationary drift, as it is characterized by smooth motion with constant speed and in some parts of the Bay is exhibits clockwise spirals due to tidal circulation. The most effective drift-correction methods should lead to drift segments becoming more contracted and increase the distinctness of spiraling due to tidal circulation. From the movement tracks (Fig. D1), it appears the BCRW-correction method used in this paper (described in Appendix B) is more effective than drift correction using satellite-based NSIDC ice motion estimates. This is also corroborated when comparing the results in Appendix B versus Appendix C, which show the BCRW-correction reduces the step length of dropped tags

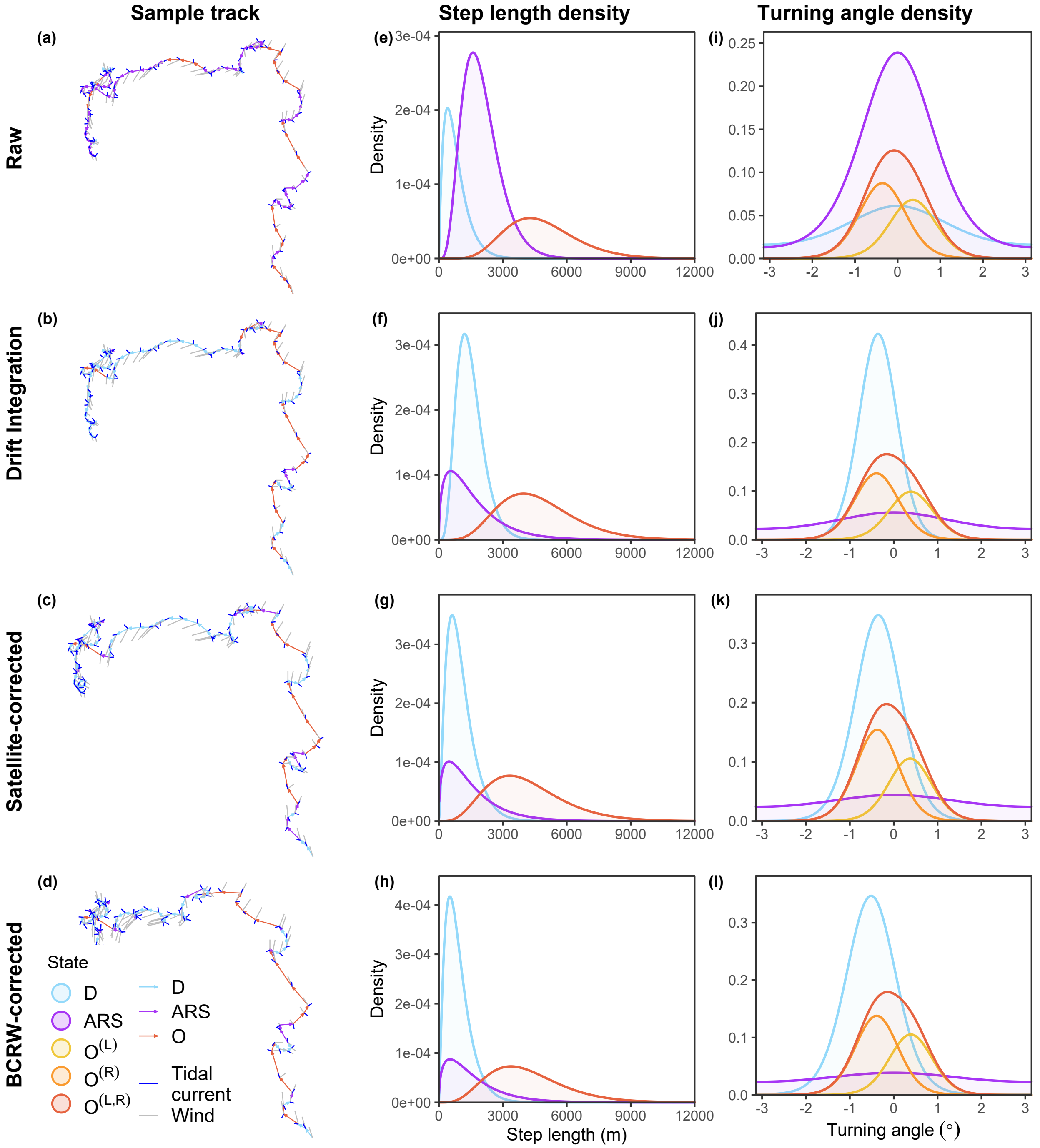

Figure D1: Predicted state characteristics of HMMs based on different drift correction methods (raw, no drift correction/integration(a, e, i), wind-based drift and tidal integration (b, f, j), satellite-based drift vector subtraction and tidal integration (c, g, k), and wind-based BCRW vector subtraction and tidal integration (d, h, l). Sample track (a-d), step length distribution for each state (e-h), and turning angle distribution for each state (i-l) are shown. D and ARS represent drift and area-restricted search, respectively, and O represent olfactory search (left, O(L), or right, O(R), relative to wind). Sample tracks in (a-d) are colored by the decoded states and show the estimated wind (gray) and tidal current (blue) velocities. All data were based on HMMs fitted to seven years of polar bear telemetry data from Western Hudson Bay, Canada.

Table D2: Confusion matrices denoting the precision rate (bold diagonal; $true.state/(true.state + false.state)$ and the false discovery rate (off-diagonal; $false.state/(true.state + false.state)$ for each combination of models (Raw, no drift correction or integration; Integ., wind-based drift and tidal integration; NSIDCc, satellite-based drift vector subtraction and tidal integration; and BCRWc, wind-based BCRW vector subtraction and tidal integration). The concurrency percentage of states predicted by the row HMM relative to the column HMM is presented on the top left of each panel. The classified states were area restricted search (ARS), drift (D), and olfactory search (O).

	BCRWc				NSIDCc				Integ.			
	**85%**	ARS	D	O								
NSIDCc	ARS	**0.80**	0.01	0.04								
	D	0.07	**0.92**	0.18								
	O	0.13	0.07	**0.78**								
	**80%**	ARS	D	O	**78%**	ARS	D	O				
Integ.	ARS	**0.84**	0.02	0.05	ARS	**0.87**	0.04	0.06				
	D	0.03	**0.87**	0.27	D	0.03	**0.80**	0.25				
	O	0.12	0.12	**0.68**	O	0.10	0.16	**0.69**				
	**45%**	ARS	D	O	**50%**	ARS	D	O	**51%**	ARS	D	O
Raw	ARS	**0.93**	0.00	0.09	ARS	**0.91**	0.00	0.12	ARS	**0.87**	0.00	0.10
	D	0.00	**0.47**	0.55	D	0.00	**0.63**	0.50	D	0.00	**0.72**	0.53
	O	0.07	0.53	**0.36**	O	0.09	0.37	**0.38**	O	0.13	0.28	**0.37**

by 29%, while the NSIDC-based correction reduced step length by 14%. Therefore, a BCRW correction is more effective than a NSIDC-based correction.

As we do not have a ground-truth of behavioral state, it is not possible to know objectively from our data which of the presented methods is most effective at state identification. We did identify a notable difference between the state identification across the methods, particularly when comparing concurrency at the level of individual steps. A key challenge when modeling movement in a moving environment is that the observed step length and orientation both depend on the bear's voluntary step length and speed as illustrated in Figure D2. To our knowledge, there is currently no statistical movement model that can simultaneously estimate effect of drift on movement and movement parameters simultaneously. For identifying the stationary drift state, the integration approach is likely very effective as there is no unknown voluntary movement. The integration method may also be effective in identifying states with no orientation bias that are significantly faster than the rate of environmental motion. For ARS, we do not predict any orientation bias, assuming the observed orientation is the voluntary orientation to calculate voluntary speed (observed vector minus drift component) may be a good approximation to model step length. However, for states where the voluntary direction is important (e.g., a BCRW), assuming the observed orientation is equal to the voluntary orientation (e.g., [McClintock and Michelot, 2018, Johnson et al., 2021]) may lead to bias both in the step length and turn angle, leading to compounding error and exaggerated misclassification. The lowest concurrency in

state identification was of the olfactory search state, followed by ARS, then drift. This is in line with the prediction the integration methods are most effective for identifying stationary behaviors, followed by unbiased behaviors, and least effective for biased behaviors. Therefore, we believe the integration method's inability to estimate the voluntary orientation, leads to misclassification of ARS, and in particular, olfactory search.

As there is no model that can simultaneously estimate the effect of drift on movement and movement parameters, there is likely no perfect solution to correct or integrate motion of environmental data, assuming it cannot be accurately and precisely measured. However, the differences among the three correction/integration methods (i.e., BCRW-based, NSIDC-based, and integration) was much smaller than between any of them and the model assuming no drift (i.e., the HMM fit to the raw locations). Therefore, any method to integrate or correct for environmental motion will likely significantly improve the models accuracy and precision – failing to consider ice drift appears to have a large effect on state prediction and may lead to significant bias.

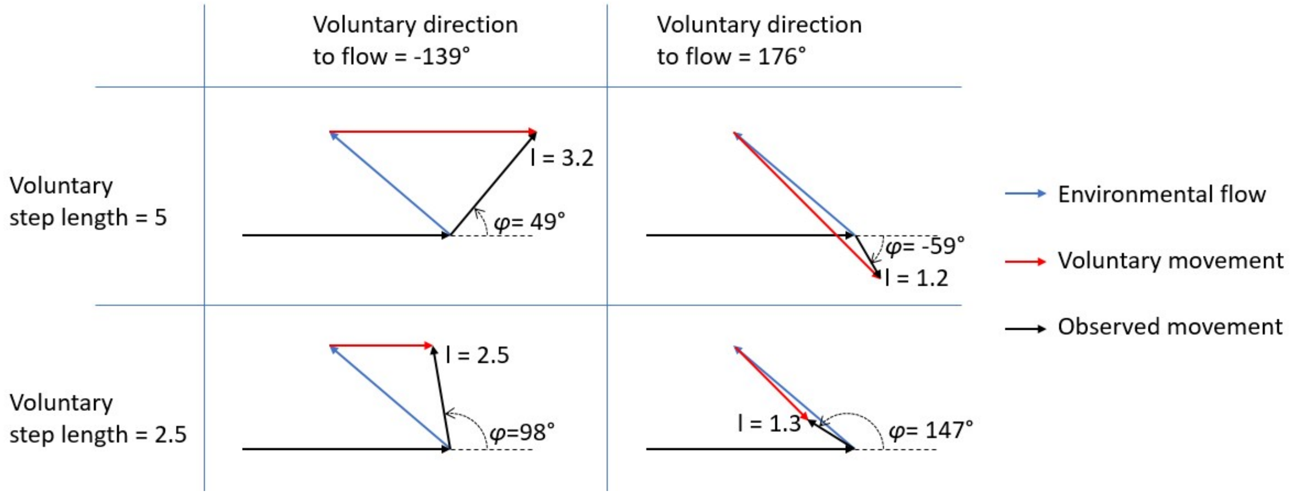

Figure D2: Schematic depicting the observed movement given the voluntary movement given the presence of environmental flow. l represents the observed step length and ϕ represents the observed turning angle. A change in either the voluntary speed or the voluntary direction changes both the observed step length and turn angle.

E Pseudo-design matrices

Pseudo-design matrix for step length in `momentuHMM`:

$$
\begin{array}{c}
\\
\mu^{(l)}_{D,t} \\
\mu^{(l)}_{ARS,t} \\
\mu^{(l)}_{O^{(L)},t} \\
\mu^{(l)}_{O^{(R)},t} \\
\sigma^{(l)}_{D,t} \\
\sigma^{(l)}_{ARS,t} \\
\sigma^{(l)}_{O^{(L)},t} \\
\sigma^{(l)}_{O^{(R)},t}
\end{array}
\begin{array}{c}
\begin{array}{ccccccc}
\beta_{1,D} & \beta_{2,D} & \beta_{1,ARS} & \beta_{1,O^{(L,R)}} & \sigma_{1,D} & \sigma_{1,ARS} & \sigma_{1,O^{(L,R)}}
\end{array} \\
\begin{pmatrix}
1 & r_t^{(tide)} & 0 & 0 & 0 & 0 & 0 \\
1 & r_t^{(tide)} & 1 & 0 & 0 & 0 & 0 \\
1 & r_t^{(tide)} & 1 & 1 & 0 & 0 & 0 \\
1 & r_t^{(tide)} & 1 & 1 & 0 & 0 & 0 \\
0 & 0 & 0 & 0 & 1 & 0 & 0 \\
0 & 0 & 0 & 0 & 0 & 1 & 0 \\
0 & 0 & 0 & 0 & 0 & 0 & 1 \\
0 & 0 & 0 & 0 & 0 & 0 & 1
\end{pmatrix}
\end{array}
\qquad (18)
$$

The column correspond to the coefficients estimated using maximum likelihood by the model, and the rows correspond to the movement parameters: step length mean ($\mu^{(l)}_{S,t}$) and standard deviation ($\sigma^{(l)}_{S,t}$). $r_t^{(tide)}$ represents tidal current speed in m s^{-1}. We ensured that $\mu^{(l)}_{D,t} < \mu^{(l)}_{ARS,t} < \mu^{(l)}_{O^{(L,R)},t}$ using the `workBounds` argument in `fitHMM` by constraining $\beta_{1,ARS}$ and $\beta_{1,O^{(L,R)}}$ to be > 0, see McClintock and Michelot [2018] for details.

Pseudo-design matrix used for turning angle:

$$
\begin{array}{c}
\\
\mu^{(\phi)}_{D,t} \\
\mu^{(\phi)}_{ARS} \\
\mu^{(\phi)}_{O^{(L)},t} \\
\mu^{(\phi)}_{O^{(R)},t} \\
\kappa^{(\phi)}_{D} \\
\kappa^{(\phi)}_{ARS} \\
\kappa^{(\phi)}_{O^{(L)}} \\
\kappa^{(\phi)}_{O^{(R)}}
\end{array}
\begin{array}{c}
\begin{array}{cccccc}
\alpha_{1,D} & \alpha_{1,O^{(L,R)}} & \alpha_{2,O^{(L,R)}} & \kappa_{1,D} & \kappa_{1,ARS} & \kappa_{1,O^{(L,R)}}
\end{array} \\
\begin{pmatrix}
\texttt{angleFormula}(\psi_t^{(tide)}, r_t^{(tide)}) & 0 & 0 & 0 & 0 & 0 \\
0 & 0 & 0 & 0 & 0 & 0 \\
0 & \psi_t^{(wind)} & \psi_t^{(wind)} + 90^\circ & 0 & 0 & 0 \\
0 & \psi_t^{(wind)} & \psi_t^{(wind)} - 90^\circ & 0 & 0 & 0 \\
0 & 0 & 0 & 1 & 0 & 0 \\
0 & 0 & 0 & 0 & 1 & 0 \\
0 & 0 & 0 & 0 & 0 & 1 \\
0 & 0 & 0 & 0 & 0 & 1
\end{pmatrix}
\end{array}
\qquad (19)
$$

`angleFormula` is a special function that can be included in design matrices to model circular parameters (e.g., mean turning angle $\mu^{(\phi)}_{D,t}$) as a circular-circular regression function of the a directional covariate and its relative strength - in this instance, tidal current direction, $\psi_t^{(tide)}$, and speed, $r_t^{(tide)}$ [Rivest and Duchesne, 2016, McClintock and Michelot, 2018]. $\psi_t^{(wind)}$ represents the direction of wind at time t relative to the animal bearing at time $t-1$.

F Diurnal and seasonal state Frequency

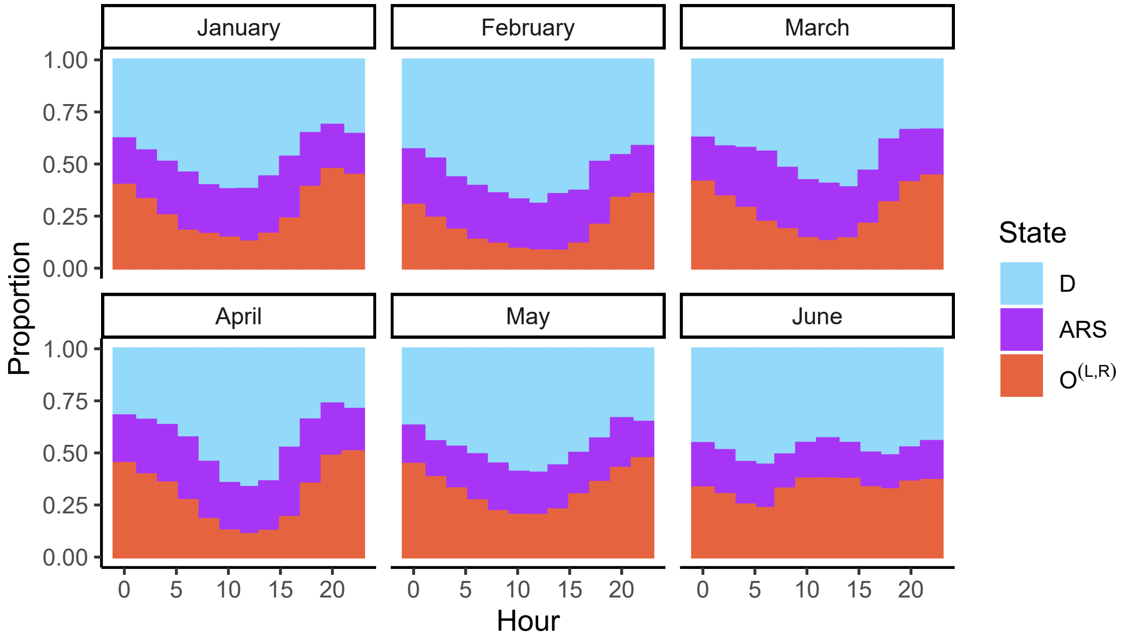

Figure F1: Proportion of states (drift D, area-restricted search ARS, and olfactory search $O^{(L,R)}$) depending on month and hour. States are based on Viterbi-decoded states.

G State distribution map

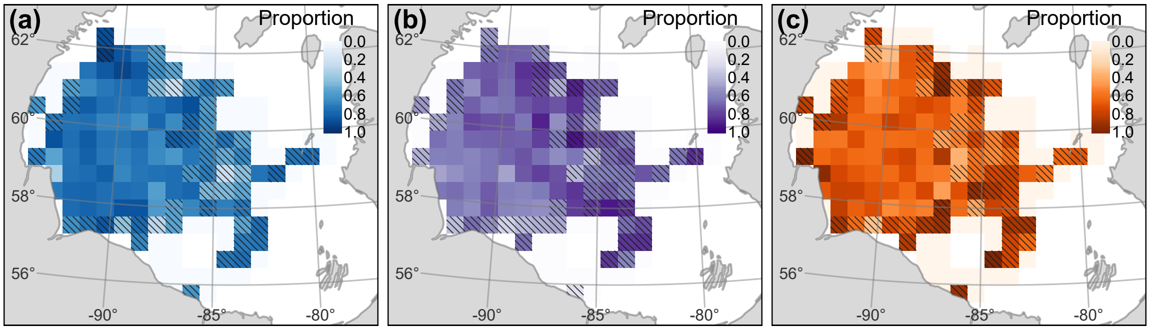

Figure G1: Distribution of predicted states. (a) proportion of bear-days for the drift state D, (b) proportion of bear-days for the area-restricted search state ARS, and (c) proportion of bear-days for the olfactory search state O. Cells with < 7 bear-days were not plotted and cells with < 21 bear days were hashed.

References

J. C. Comiso. Bootstrap Sea Ice Concentrations from Nimbus-7 SMMR and DMSP SSM/I-SSMIS, Version 3, 2017. URL `https://nsidc.org/data/nsidc-0079/versions/3`.

Hans Hersbach, Bill Bell, Paul Berrisford, Shoji Hirahara, András Horányi, Joaquín Muñoz-Sabater, Julien Nicolas, Carole Peubey, Raluca Radu, Dinand Schepers, Adrian Simmons, Cornel Soci, Saleh Abdalla, Xavier Abellan, Gianpaolo Balsamo, Peter Bechtold, Gionata Biavati, Jean Bidlot, Massimo Bonavita, Giovanna Chiara, Per Dahlgren, Dick Dee, Michail Diamantakis, Rossana Dragani, Johannes Flemming, Richard Forbes, Manuel Fuentes, Alan Geer, Leo Haimberger, Sean Healy, Robin J. Hogan, Elías Hólm, Marta Janisková, Sarah Keeley, Patrick Laloyaux, Philippe Lopez, Cristina Lupu, Gabor Radnoti, Patricia Rosnay, Iryna Rozum, Freja Vamborg, Sebastien Villaume, and Jean-Noël Thépaut. The ERA5 global reanalysis. *Quarterly Journal of the Royal Meteorological Society*, 146(730):1999–2049, jul 2020. ISSN 0035-9009. doi: 10.1002/qj.3803. URL `https://onlinelibrary.wiley.com/doi/abs/10.1002/qj.3803`.

Robert J Hijmans and Jacob van Etten. *Package "raster". Geographic data analysis and modeling*, 2016.

IOC, IHO, and BODC. Centenary edition of the GEBCO Digital Atlas. Vol. General Bathy- metric Chart of the Oceans., 2003.

Devin Johnson, Noel Pelland, and Jeremy Sterling. A continuous-time semi-Markov

model for animal movement in a dynamic environment. *The Annals of Applied Statistics*, 15(2):797–812, jun 2021. ISSN 1932-6157. doi: 10.1214/20-AOAS1408. URL https://projecteuclid.org/journals/annals-of-applied-statistics/volume-15/issue-2/A-continuous-time-semi-Markov-model-for-animal-movement-in/10.1214/20-AOAS1408.full.

Devin S Johnson and Josh M. London. *crawl: an R package for fitting continuous-time correlated random walk models to animal movement data.*, 2018.

Devin S Johnson, Joshua M. London, Mary-Anne Lea, and John W. Durban. Continuous-time correlated random walk model for animal telemetry data. *Ecology*, 89(5):1208–1215, 5 2008. ISSN 0012-9658. doi: 10.1890/07-1032.1.

Dan E Kelley, Clark Richards, and Chantelle Layton. oce: an R package for oceanographic analysis, 2022.

Roland Langrock, Ruth King, Jason Matthiopoulos, Len Thomas, Daniel Fortin, Juan Manuel Morales, and Roland Angrock. Flexible and practical modeling of animal telemetry data: hidden Markov models and extensions. *Ecology*, 93(11):2336–2342, 11 2012. ISSN 00129658. doi: 10.1890/11-2241.1. URL http://eprints.gla.ac.uk/78766/http://eprints.gla.ac.uk.

Brett T McClintock and Théo Michelot. momentuHMM: R package for generalized hidden Markov models of animal movement. *Methods in Ecology and Evolution*, 9(6):1518–1530, 2018. ISSN 2041210X. doi: 10.1111/2041-210X.12995.

Brett T McClintock, Roland Langrock, Olivier Gimenez, Emmanuelle Cam, David L. Borchers, Richard Glennie, and Toby A Patterson. Uncovering ecological state dynamics with hidden Markov models. *Ecology Letters*, 23(12):1878–1903, 12 2020. ISSN 1461-023X. doi: 10.1111/ele.13610. URL http://arxiv.org/abs/2002.10497https://onlinelibrary.wiley.com/doi/10.1111/ele.13610.

Louis-paul Rivest and Thierry Duchesne. A general angular regression model for the analysis. *Applied Statistics*, 65(3):445–463, 2016. doi: 10.1111/rssc.12124.

RStudio Team. *RStudio: Integrated Development Environment for R.* RStudio, PBC, Boston, MA, 2021. URL http://www.rstudio.com/.

Pierre St-Laurent, F. J. Saucier, and J. F. Dumais. On the modification of tides in a seasonally ice-covered sea. *Journal of Geophysical Research: Oceans*, 113(11):C11014, 2008. ISSN 21699291. doi: 10.1029/2007JC004614.

Ron R Togunov, Natasha J. Klappstein, Nicholas J. Lunn, Andrew E Derocher, and Marie Auger-Méthé. Opportunistic evaluation of modelled sea ice drift using passively drifting telemetry collars in Hudson Bay, Canada. *The Cryosphere*, 14(6):1937–1950, 2020. ISSN 1994-0416. doi: 10.5194/tc-2020-26.

Ron R Togunov, Andrew E Derocher, Nicholas J Lunn, and Marie Auger-Méthé. Characterising menotactic behaviours in movement data using hidden Markov models. *Methods in Ecology and Evolution*, 12:1984–1998, 2021. doi: https://doi.org/10.1111/2041-210X.13681. URL https://besjournals.onlinelibrary.wiley.com/doi/10.1111/2041-210X.13681.

Mark A. Tschudi, Charles W. Fowler, James A. Maslanik, and Julienne Stroeve. Tracking the movement and changing surface characteristics of Arctic sea ice. *IEEE Journal of Selected Topics in Applied Earth Observations and Remote Sensing*, 3(4):536–540, 2010. ISSN 21511535. doi: 10.1109/JSTARS.2010.2048305.

Mark A. Tschudi, W. N. Meier, J. S. Stewart, C. Fowler, and J. Maslanik. *Polar Pathfinder Daily 25 km EASE-Grid Sea Ice Motion Vectors, Version 4*. Boulder, Colorado USA, 2019.

Mark A. Tschudi, Walter N. Meier, and J. Scott Stewart. An enhancement to sea ice motion and age products. *The Cryosphere*, 14:1519–1536, 2020. ISSN 1994-0440. doi: 10.5194/tc-2019-40.
